# Integrating enzyme-nanoparticles bring new prospects for the diagnosis and treatment of immune dysregulation in periodontitis

**DOI:** 10.3389/fcimb.2024.1494651

**Published:** 2024-11-01

**Authors:** Qianqian Zhang, Zhiyi Wang, Shijiao Shen, Junzhe Wang, Jun Cao, Yongqiang Deng, He Meng, Lin Ma

**Affiliations:** ^1^ Department of Stomatology, Shenzhen University General Hospital, Shenzhen University, Shenzhen, Guangdong, China; ^2^ Institute of Stomatological Research, Shenzhen University, Shenzhen, Guangdong, China; ^3^ School of Stomatology, Shenzhen University, Shenzhen, Guangdong, China

**Keywords:** enzyme-responsive nanoparticles, nanozymes, periodontitis biomarkers, biosensors, fluorescent probes, inflammatory

## Abstract

Enzymes play a significant role in mediating inflammatory and immune responses in periodontitis. Effective diagnosis, timely treatment, and continuous management of periodontal enzymes are essential to prevent undesirable consequences; however, this remains a significant challenge. Nanoparticles (NPs) have attracted significant attention in biomedicine because of their advantageous nanosized effects. NPs are conjugated with specific enzyme substrates at responsive sites that are triggered by periodontitis enzyme biomarkers, leading to functional or characteristic changes. In contrast, NPs with enzyme-mimetic activities exhibit catalytic activity, effectively destroying pathogenic biofilms and modulating the immune response in periodontitis. The unique properties of enzyme-targeting NPs have enabled the development of biosensors and fluorescent probes capable of identifying enzyme biomarkers associated with periodontitis. Enzyme-responsive and enzyme-mimetic NPs both exert therapeutic applications in the treatment of periodontitis. In this review, we provide a comprehensive overview of the enzymes associated with periodontitis, the mechanisms of enzyme-responsive and enzyme-mimetic NPs, recent advancements in the use of NPs for detecting these enzymes, and the therapeutic applications of NPs in targeting or mimicking enzyme functions. We also discuss the challenges and prospects of using NPs in the diagnosis and treatment of periodontitis.

## 1 Introduction

Periodontitis is a chronic inflammatory disease associated with dysbiotic microbiota (Cennamo et al., 2024). It may induce periodontal attachment destruction, bone resorption, and eventual tooth loss (Garcia et al., 2018; Gharbi et al., 2019; Caldeira et al., 2021). This disease is a major contributor to the global burden of chronic conditions and affects approximately 20%–50% of the population worldwide (Kc et al., 2020). Periodontitis is the sixth most prevalent disease globally (Arias-Bujanda et al., 2020). According to a report from World Health Organization, severe periodontal disease affects approximately 19% of the global adult population, accounting for over 1 billion cases worldwide (Nazir et al., 2020; Hooshiar et al., 2024). Epidemiological studies reveal the highest incidence in older adults (82%), followed by adults (73%) and adolescents (59%) (Liu C. et al., 2022; Hooshiar et al., 2024). Periodontitis significantly reduced the quality of patients’ life and increased the risk of systemic diseases (e.g. diabetes, cardiovascular diseases, rheumatoid arthritis, and cancer) and adverse pregnancy outcomes (Hartenbach et al., 2020; Kc et al., 2020; Toczewska et al., 2020; Blanco-Pintos et al., 2023; Cennamo et al., 2024). Research has shown that oral microbiota play a role in the development of systemic diseases. In immunocompromised populations, opportunistic or pathogenic bacteria from the oral cavity can breach the oral mucosal barrier and enter the bloodstream, potentially triggering abnormal immune and metabolic responses both locally and systemically (Hartenbach et al., 2020; Pei et al., 2020). Periodontal bacteria, inflammatory immune responses, lifestyle, and medical conditions are the common risk factors for periodontitis (Hartenbach et al., 2020). Periodontopathogenic bacteria in the gingival crevices are considered the primary cause of periodontitis (Gharbi et al., 2019). These bacteria can lead to chronic inflammation and plaque formation in the periodontal region, which evolves into hardened bacterial films, enlarging the gingival sulcus, and causing tooth loss.

Periodontal disease is one of the most destructive dental pathologies (Kim et al., 2023), and accurate diagnosis is essential for effective treatment, given its global prevalence (Koller et al., 2021). Traditionally, chronic periodontal disease was diagnosed through clinical measurements and radiographic assessments such as probing depth, clinical attachment level, plaque index, bleeding scores, and bone loss severity (Arias-Bujanda et al., 2019). Although effective, these methods do not accurately reflect the underlying inflammatory response and have the following limitations (Arias-Bujanda et al., 2020): (1) They are time-consuming, prone to measurement error, subjective, and poorly tolerated by patients. (2) These parameters only assess the severity, not the current activity or future course of the disease, leading to delayed diagnoses, late interventions, and worsening of the condition (Cennamo et al., 2024). Therefore, supplementary diagnostic approaches are required. Identifying biological signatures that highlight risk, track disease progression, monitor health status, and indicate treatment outcomes offers significant clinical advantages (Kc et al., 2020). Timely and accurate diagnosis of periodontal conditions is crucial due to potential complications from tissue and bone loss (Blanco-Pintos et al., 2023). Non-invasive and early diagnostic methods are essential to supplement or replace conventional clinical measurements (Arias-Bujanda et al., 2019; Kim et al., 2023). Real-time monitoring of chronic inflammation is critical for preventing periodontal diseases (Kim et al., 2023). Traditional treatments such as mechanical debridement, antibiotic therapy, and surgical interventions have limitations, particularly in tissue regeneration (Zhou et al., 2021; Osuna-Ramos et al., 2023; Hosseini Hooshiar et al., 2024). Mechanical debridement often causes discomfort, bleeding, and gingival damage (Hosseini Hooshiar et al., 2024). Antibiotic therapy has diminishing effects on the oral environment, requiring frequent use and encountering issues such as drug resistance, microbial imbalance, and limited tissue repair capacity (Zhou et al., 2021; Hosseini Hooshiar et al., 2024). Thus, there is a need for antibiotic-free, non-invasive, rapid, and effective treatments for periodontitis (Zhou et al., 2021; Hosseini Hooshiar et al., 2024). New approaches should enhance biofilm elimination and promote tissue regeneration without the adverse effects of the traditional methods.

Recent studies have advanced our understanding of periodontal disease pathways using non-invasively accessible samples such as blood, saliva, gingival crevicular fluid (GCF), and oral rinse. The latest classification of periodontal and peri-implant diseases highlights the potential of biomarkers for improving early diagnosis (Hartenbach et al., 2020; Blanco-Pintos et al., 2023). Researchers are investigating biochemical biomarkers in the GCF and saliva to predict, diagnose, and monitor periodontitis (Blanco-Pintos et al., 2023), aiding in determining disease severity and evaluating treatment outcomes (Toczewska et al., 2020). Recognized biomarkers for periodontitis include enzymes, proteins, inflammatory factors, collagen and bone resorption products, and DNA from host or bacterial sources (Pei et al., 2020; Katsiki et al., 2021). These biomarkers, along with the oral microflora and metabolites, can facilitate patient categorization, disease tracking, anticipatory diagnostics, and patient-focused prevention (Pei et al., 2020). Enzymes are essential in living organisms and mediate nearly all chemical reactions involved in key cellular processes, such as signal transduction, DNA replication, immune responses, and metabolism. They play crucial roles in metabolic and biological functions (Lin et al., 2016; Kapalatiya et al., 2022). Enzymes associated with periodontal disease not only reveal the progression of periodontitis but can also achieve therapeutic effects through their regulation. Early diagnosis enables targeted preventive measures, such as improved oral hygiene, correct brushing techniques, a healthy diet, a healthier lifestyle, and regular check-ups. Understanding and characterizing periodontitis biomarkers is essential for developing public healthcare strategies and personalized treatment approaches (Pei et al., 2020). Nanoparticles (NPs) offer promising advancements in the diagnosis and treatment of periodontitis, effectively addressing these traditional limitations owing to their unique properties (Hooshiar et al., 2024; Zhang S. et al., 2024). NPs have rapidly advanced bioassay methodologies, and NP-based detection strategies are gaining popularity as quick and efficient diagnostic assays (Hooshiar et al., 2024). The application of NPs in the treatment of periodontitis has evolved from a material-oriented approach to a function-oriented approach. These advanced NPs exhibit antibacterial and anti-inflammatory properties, emphasizing their multifunctional and synergistic effects (Hosseini Hooshiar et al., 2024). The rapid growth in nanotechnology and biotechnology offers a wealth of opportunities for combining enzymes with different types of NPs. The construction of NP-incorporated enzymes integrates the specific recognition and biocatalytic properties of enzymes with the attractive electronic, optical, magnetic, and catalytic properties of NPs (Lin et al., 2016).

Therefore, in this review, we summarize the various enzyme biomarkers associated with periodontitis and provide an overview of the mechanisms of NP-targeted and mimicked enzymes. Additionally, we describe the diagnostic and therapeutic applications of NPs in periodontitis.

## 2 Enzymes biomarkers in periodontitis

Dysregulated enzyme activity is often linked to various diseases and disorders, making enzymes attractive therapeutic targets due to their high substrate specificity and precise biological interactions (Kapalatiya et al., 2022). Additionally, detecting enzyme activity is a valuable diagnostic tool (de la Rica et al., 2012), as abnormal enzyme expression is a hallmark of many diseases (Lin et al., 2016). Furthermore, the remarkable catalytic efficiency of enzymes can be leveraged to enhance the signals generated during the recognition of specific analytes (de la Rica et al., 2012).

**Figure 1:**
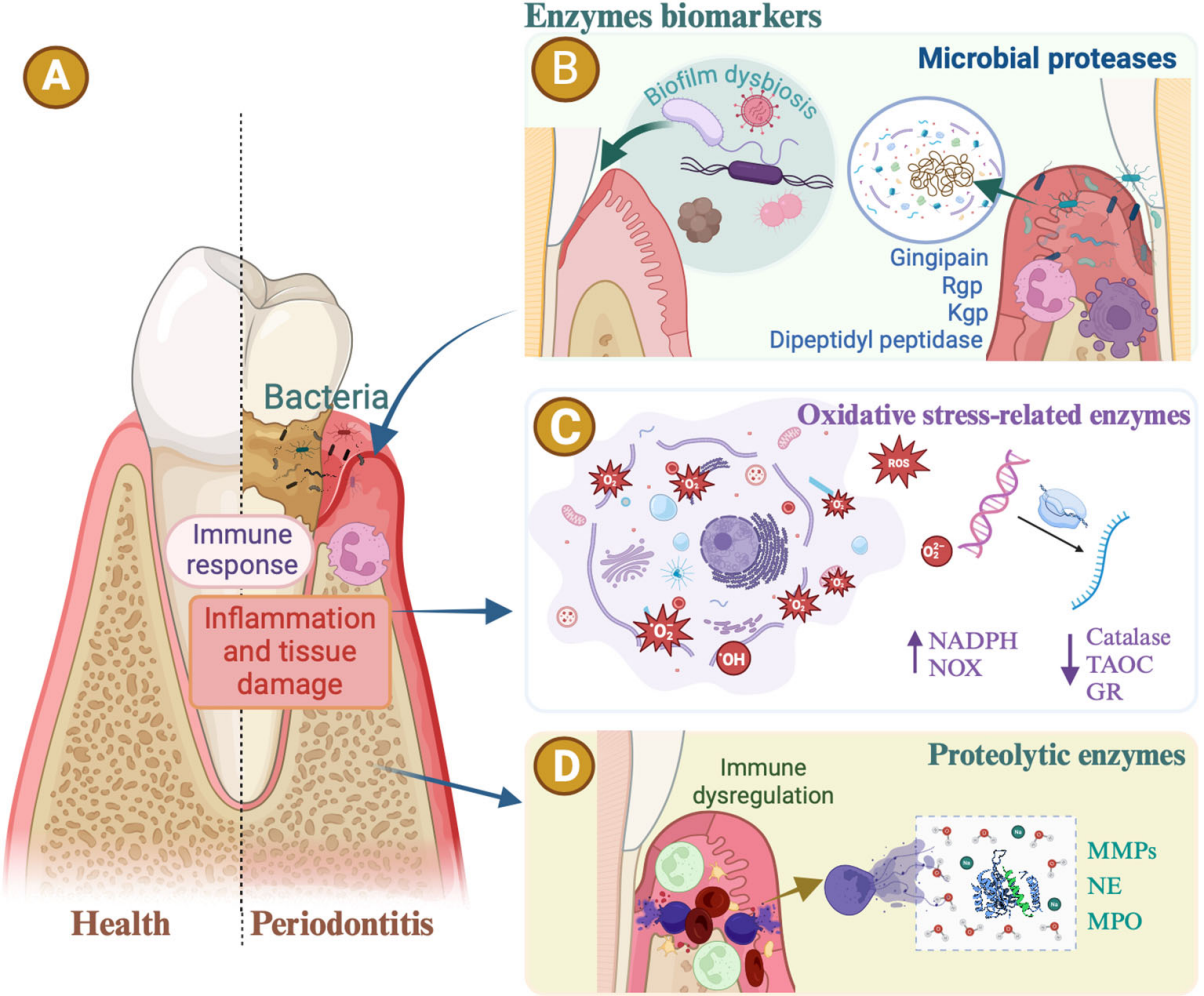
Enzymes biomarkers in periodontitis. **(A)** The periodontal bacteria. Inflammatory immune responses are all common risk factors for periodontitis. Enzymes associated with periodontal disease reveal the progression of periodontitis. **(B)** Oral microbial proteases. Gingipain secreted by *Porphyromonas gingivalis*, including arginine protease (Rgp) and lysine gingipain (Kgp), serve as virulence factors, hydrolyzing host proteins, increasing bleeding tendency, activating inflammation, and causing alveolar bone loss. The dipeptidyl peptidase (DPP) orthologs are specifically distributed in anaerobic oral rods. DPP activities in dental plaque are potent biomarkers indicating the presence of periodontopathic bacteria and disease activity. **(C)** Oxidative stress-related enzymes. Periodontitis is intricately linked with oxidative-reductive imbalance and oxidative stress. Evidence suggests that patients with periodontitis exhibit elevated levels of oxidative stress markers and reduced levels of antioxidants in their serum or saliva. The activity of nicotinamide adenine dinucleotide phosphate (NADPH) oxidase, or non-phagocytic cell oxidase (NOX) enzyme complexes, increases in patients with periodontal disease. Enzymatic antioxidants associated with periodontitis include catalase, glutathione reductase (GR), and total antioxidant capacity (TAOC). **(D)** Proteolytic enzymes. The levels of matrix metalloproteinases (MMPs), neutrophil elastase (NE), and myeloperoxidase (MPO) increase in periodontitis, suggesting these enzymes as potential markers of periodontal inflammation.

Subgingival gram-negative bacteria trigger host tissue degradation and a robust inflammatory response mediated by polymorphonuclear leukocytes (PMNs), macrophages, and lymphocytes (**Figure 1A**). These cells release proteinases, cytokines, and prostaglandins, leading to the destruction of soft and mineralized tissues and are crucial biomarkers in the disease’s initiation, progression, and modulation (Caldeira et al., 2021). A study by the National Institute of Dental and Craniofacial Research highlighted that severe periodontitis is associated with an excessive immune response, characterized by neutrophil accumulation, reactive oxygen species (ROS) production, and neutrophil extracellular traps (Sies and Jones, 2020; Silva et al., 2021). Biomarkers of periodontitis are categorized based on the affected tissue or disease progression stage. Some researchers categorize these biomarkers as markers (products) of connective tissue degradation, soft tissue inflammation, and alveolar bone loss (Cennamo et al., 2024). This review summarized enzymes biomarkers associated with periodontitis, covering the main biological phases of periodontitis (Arias-Bujanda et al., 2019), such as microbial proteases, oxidative stress-related enzymes, proteolytic enzymes, and other enzymes (**Figure 1**).

### 2.1 Oral microbial proteases

The oral microbiome, comprising over 1,000 bacterial species, plays a crucial role in human health (Gregorczyk-Maga et al., 2023). Periodontitis results from microbial dysbiosis, leading to an overactive host immune response and subsequent tissue destruction. Dysbiosis alters protein and metabolite expression, producing specific molecular biomarker profiles that are detectable in oral fluids (Blanco-Pintos et al., 2023) (**Figure 1B**). Clinical indices, subgingival microbiota, and GCF biochemical compounds differ significantly between patients with periodontitis and healthy individuals, highlighting host-microbe interactions in this disease (Arias-Bujanda et al., 2019). Pathogenic microbes, especially anaerobic gram-negative bacteria, trigger periodontitis through polymicrobial infection in subgingival plaques (Hirai et al., 2020; Malone et al., 2021). The composition of the subgingival microflora differs significantly between healthy and diseased sites (Genco et al., 2019).

*Porphyromonas gingivalis(P.g)*, is a keystone pathogen associated with periodontitis and various systemic diseases (Hirai et al., 2020). Gingipain secreted by *P. g*, including arginine protease (Rgp) and lysine gingipain (Kgp), serve as virulence factors, hydrolyzing host proteins, increasing bleeding tendency, activating inflammation, and causing alveolar bone loss (Koller et al., 2021; Liu et al., 2021). High serum IgG levels against *P.g.* correlate with the progression (Hirai et al., 2020). Hirai et al. identified RgpA as the best antigen for screening patients with periodontitis, suggesting its potential as a rapid diagnostic kit (Hirai et al., 2020). In addition to *P.* gingivalis, *Treponema denticola* (*T*.*d*.), another periodontal pathogen, contributes to dysbiosis, chronic inflammation, extracellular matrix (ECM) remodeling, and tissue destruction (Malone et al., 2021). *T.d*. Microbial proteinases potentiate the activation of matrix metalloproteinases (MMPs) via oxidative pathways. TD-derived proteases and virulence factors derived from *T.d.* activate proMMPs (Gupta et al., 2023). *T.d.* affects filamentous actin abundance in gingival fibroblasts and epithelial cells (Malone et al., 2021). Its outer membrane protein induces the release of cathepsin G and elastase from PMNs (Gupta et al., 2023). Dysbiotic pathogens such as *P.g, Fusobacterium nucleatum*(*F*.*n*)*, and Actinobacillus actinomycetemcomitans*(*A.c*) can induce actin reorganization, which facilitates periodontitis (Malone et al., 2021). *P*.*g. F*.*n*. *A.c.* produces microbial proteases that activate latent MMPs (MMP-1, MMP-8, and MMP-9) and stimulate their production in inflammatory cells, fibroblasts, epithelial cells, and periodontal ligament cells (Gupta et al., 2023). Yuko et al. revealed that dipeptidyl peptidase (DPP) orthologs are specifically distributed in anaerobic oral rods, indicating a potential link between periodontopathic bacterial DPPs and periodontal and systemic diseases. DPP activity in dental plaque is a potent biomarker that indicates the presence of periodontopathic bacteria and disease activity. DPP4, DPP5, DPP7, and DPP11 are found in anaerobic periodontopathic rods, and their activities vary among individual specimens. These DPPs are crucial for energy production in *P.g*, an asaccharolytic bacterium that causes periodontal diseases. DPP4 activity, primarily human-derived, was predominantly observed in saliva samples. These results indicate that P.g.-DPPs, especially DPP4, are generally distributed among the anaerobic rods in subgingival dental plaque (Ohara-Nemoto et al., 2018).

Researchers have focused on understanding microbiome activity and characterizing biomarkers to improve diagnostic accuracy, monitor disease progression, and evaluate treatment efficacy.

### 2.2 Oxidative stress-related enzymes

Periodontitis is intricately linked to oxidative-reductive imbalance (Toczewska et al., 2020) and oxidative stress (**Figure 1C**), a key pathophysiological mechanism at the molecular scale (Baima et al., 2021). Oxidative stress is the hallmark of periodontitis (Cao et al., 2024). Numerous studies have shown that oxidative stress directly contributes to the degradation of extracellular matrix components in periodontal tissues, including collagen, elastin, proteoglycans, and glycosaminoglycans (e.g., hyaluronic acid). This degradation leads to the destruction of the periodontal attachment apparatus and the release of lysosomal enzymes, further contributing to local tissue destruction (Toczewska et al., 2020). Increased production of ROS results from the inflammatory infiltration of PMNs as a host response to microbial invasion (Cao et al., 2024). Neutrophil accumulation leads to increased ROS levels and oxidative stress (Cao et al., 2024). Stimulation of neutrophils by periodontopathogens triggers a “respiratory burst,” resulting in increased formation of ROS and nitrogen species such as hypochlorous acid, superoxide anion, and hydrogen peroxide (Toczewska et al., 2020). The effects of ROS are modulated *in vivo* by the antioxidant defense systems (Gharbi et al., 2019).

Evidence suggests that patients with periodontitis exhibit elevated levels of oxidative stress markers and reduced levels of antioxidants in the serum or saliva (Cao et al., 2024) (**Figure 1C**). Oxidative stress-related metabolites have frequently been investigated in targeted GCF analyses (Baima et al., 2021). Thomas et al. reported that increased lipid peroxidation levels in the GCF reflect heightened ROS damage in the periodontal tissues (Gharbi et al., 2019). The activity of nicotinamide adenine dinucleotide phosphate (NADPH) oxidase, or non-phagocytic cell oxidase (NOX) enzyme complexes (Zeng et al., 2019), increases in patients with periodontal disease, leading to enhanced production of free radicals and serving as a significant source of pro-inflammatory cytokines. Specifically, NADPH oxidase NOX_2_ plays a crucial role in periodontal pathology (Toczewska et al., 2020). Enzymatic antioxidants associated with periodontitis include catalase (CAT), glutathione reductase (GR), and total antioxidant capacity. CAT, an enzyme in the oxidoreductase group, converts hydrogen peroxide (H_2_O_2_) into water and oxygen, making it one of the most powerful antioxidants (Yilgor and Demir, 2024). Gharbi et al. showed a statistically significant decrease in erythrocyte CAT activity, which is consistent with the findings of Thomas et al., suggesting reduced CAT levels in patients with advanced-stage periodontal disease. Conversely, Panjamurthy et al. found elevated levels of CAT and other enzymatic antioxidants, such as superoxide dismutase (SOD), in patients compared to controls (Gharbi et al., 2019). The GR is a crucial antioxidant enzyme. Reduced glutathione serves as a vital intracellular antioxidant for ROS detoxification and is an indicator of antioxidant defense status (Baima et al., 2021). Reduced glutathione removes reactive oxygen molecules from the cells and decomposes H_2_O_2_ into H_2_O and O_2_. It plays multiple biological roles, including preventing oxidative stress, removing hydroperoxides, detoxifying, and stabilizing biological membranes. Chapple et al. reported lower concentrations of glutathione in the serum and GCF of patients with periodontal disease, findings that align with those of Ahmed et al. (Gharbi et al., 2019) and other studies. The antioxidant capacity (TAC) of the GCF, saliva, and blood is crucial for periodontitis. Toczewska et al. observed significantly reduced TAC in GCF from periodontal pockets compared to gingival fissures, suggesting a negative impact of reduced TAC on periodontal health. Chapple et al. found that treatment significantly improved the TAC in patients with GCF. Most studies report significantly reduced salivary TAC in patients with periodontitis using the ABTS oxidation method. A meta-analysis by Chen et al. involving 556 participants confirmed this significant reduction of salivary TAC in patients with periodontitis compared with healthy people (p = 0.003; inhomogeneity index, 98.3%). Toczewska et al. also observed a reduced TAC in non-stimulated saliva, with only one study showing no significant differences of salivary TAC between periodontitis patients and healthy people. Additionally, significant decreases in TAC in the peripheral blood of patients with periodontitis have been demonstrated using oxidation methods. Only two studies found no effect of periodontal diagnosis on TAC in blood serum or plasma, likely due to differing testing methodologies (Toczewska et al., 2020).

Ghezzi (2020) classified the oxidative stress biomarkers into five subgroups. Type 0 biomarkers involve the direct measurement of ROS *in vivo* in patients, while Type 1 biomarkers, the most commonly used indicators, reflect oxidative damage to lipids, proteins, or nucleic acids, including GR. Type 2 biomarkers are molecules involved in the activation of biochemical pathways such as CAT, which can lead to ROS formation. Type 3 biomarkers encompass host factors such as small molecular weight antioxidants and antioxidant enzymes, including NADPH. Type 4 biomarkers measure genetic factors and mutations that may alter an individual’s susceptibility to oxidative stress (Ghezzi, 2020; Demirci-Çekiç et al., 2022).

### 2.3 Proteolytic enzymes

Saliva contains various host proteases, including cysteine cathepsins, serine cathepsins, aspartic cathepsins, kallikreins, metalloproteases (MMP-1, MMP-3, MMP-7, MMP-8, and MMP-9), chymotrypsin-like proteinase 3 (PRTN3), and dipeptidyl peptidase (DPPIV/CD26). These proteolytic enzymes impact tissue integrity and repair, functioning as both indicators and diagnostic markers for oral diseases (**Figure 1D**) (Koller et al., 2021). Neutrophils play a crucial role in maintaining host-bacterial homeostasis in periodontitis by migrating in greater numbers to inflamed gingival tissues and accumulating in the gingival pocket epithelium and nearby connective tissues (Kayar et al., 2021; Omer-Cihangir et al., 2021). The rapid degranulation and enzyme release of neutrophils during microbial phagocytosis support a cascade that is key to the pathophysiology of periodontitis. They are the main source of collagenases and other proteinases responsible for matrix degradation in the diseased periodontium, providing MMPs and serine proteases (Gupta et al., 2023). Enzymatic activity is essential for neutrophil defense and the progression of periodontal disease from gingivitis to periodontitis (Omer-Cihangir et al., 2021). Proteolytic enzymes, as mediators of extracellular matrix degradation, are promising diagnostic markers for these conditions.

Koller et al. suggested that dysregulated proteases are linked to oral disease status (Koller et al., 2021). In human periodontal ligament damage, host-derived cysteine and serine proteases, including cathepsins and elastases, are expressed sequentially in response to inflammation or microbial invasion. These proteases stimulate mesenchymal proliferation, epithelial cell migration and wound closure (Gürsoy et al., 2020). According to Arias-Bujanda et al., MMP-8, elastase, cathepsin, and trypsin are the most studied biomarkers of periodontal tissue degradation (Arias-Bujanda et al., 2019). Silbereisen et al. found that MMP-8 and other neutrophil-derived molecules, such as tissue inhibitors of metalloproteinase 1, myeloperoxidase (MPO), and neutrophil elastase (NE), are abundant in saliva, with mean concentrations of over 700 ng/mL. Protein concentrations between 70 and 700 ng/mL are the detection limit for reliable quantification by mass spectrometry (MS) applications in plasma (Silbereisen et al., 2020). Neutrophils are the main source of host proteolytic enzymes, including NE, MPO, cathepsins, trypsin, and serine proteases (Silbereisen et al., 2020). GCF levels of MPO, beta-glucuronidase (BGD), and NE increase in periodontitis, suggesting that these enzymes are potential markers of periodontal inflammation (Omer-Cihangir et al., 2021). The median sensitivity and specificity were 76.7% and 92.0% for MMP-8, 74.6% and 81.1% for NE, and 72.8% and 67.3% for cathepsin (Arias-Bujanda et al., 2019). Arias-Bujanda’s review highlighted MMP-8 and NE as the most researched biomarkers (Arias-Bujanda et al., 2019). MMP-8 and NE are the only enzymes studied for their predictive value in periodontal disease progression and treatment outcomes (Kayar et al., 2020). MMP-8 and NE activities decrease after periodontal treatment (Gürsoy et al., 2020). NE is crucial for initiating neutrophil extracellular trap formation, and works with MPO to drive chromatin de-condensation (Omer-Cihangir et al., 2021). This serine protease, released during phagocytosis and cell lysis, acts against elastin, collagen, fibronectin, hemoglobin (Hb), laminin 1, proteoglycans, and thrombospondins, thereby facilitating neutrophil transmigration. Excessive NE secretion leads to extracellular matrix degradation (Omer-Cihangir et al., 2021). In addition to NE, the serine protease group includes cathepsin G (Cat G) and proteinase 3 (PR3), both of which are stored in high concentrations in the azurophilic granules of neutrophils. Upon neutrophil activation, Cat G is released to degrade engulfed pathogens intracellularly and also facilitates the extracellular breakdown of structural ECM components and other immune mediators (Gramegna et al., 2017). PR3, similar to NE, performs a comparable biological role by proteolytically degrading and activating IL-8, thereby enhancing its chemoattractant effect on neutrophils (Gramegna et al., 2017). Molecules such as MPO and BGD are proteolytic enzymes released from neutrophil azurophilic granules into the extracellular area following neutrophil stimulation (Omer-Cihangir et al., 2021) and are promising GCF biomarkers for diagnosing periodontitis (Arias-Bujanda et al., 2019). MPO, a haem protein, constitutes at least 5% of the dry weight of leukocytes. It generates reactive oxygen species in the peroxidation and halogenation cycles and exhibits bactericidal activity with hydrogen peroxide. BGD, a lysosomal acid hydrolase, degrades proteoglycans in connective tissue (Omer-Cihangir et al., 2021). Macrophages and fibroblasts also accumulate in the altered periodontium, producing cytokines, MMPs, and proteolytic enzymes, leading to the destruction of the periodontal attachment (Hartenbach et al., 2020). Tissue Inhibitor of Metalloproteinases-1 (TIMP-1), produced by fibroblast or mesenchymal cells, inhibits MMP-8 expression NE and MPO activate MMP-8 and inactivate TIMP-1 (Silbereisen et al., 2020).

#### 2.3.1 MMP-8

MMPs, particularly MMP-8, play a crucial role in periodontitis and are closely associated with periodontal status (Arias-Bujanda et al., 2019). Upon selective degranulation of PMNs, latent MMPs are activated by ROS or proteolytic cleavage. In saliva, the predominant forms of MMPs are pro-forms or inactive forms (Koller et al., 2021; Gupta et al., 2023). MMP-8 is primarily expressed by neutrophils and is essential for wound healing and tissue regeneration (Silbereisen et al., 2020). It is present in saliva and serum, making it a promising biomarker that is often detectable before radiographic and clinical signs of gingival and alveolar bone damage (Cennamo et al., 2024). Studies since 2005 have demonstrated the association between MMP-8 and various periodontal diseases, highlighting its predictive capability (Arias-Bujanda et al., 2019; Arias-Bujanda et al., 2020). Several diagnostic kits for periodontitis and peri-implantitis based on MMP-8 have been commercialized (Arias-Bujanda et al., 2019). MMP-8’s good sensitivity and excellent specificity make it the most effective biomarker for diagnosing periodontitis in systemically healthy participants (Arias-Bujanda et al., 2019), with a median sensitivity and specificity of 76.7% and 92.0%, respectively (Arias-Bujanda et al., 2020). In a meta-analysis, MMP-8 was identified as the second-best salivary biomarker, with an estimated sensitivity of 72.5% (Arias-Bujanda et al., 2020).

MMP-8 exists in various molecular forms and activity stages, including latent proforms (proMMP-8), active forms (aMMP-8), and fragmented forms (Arias-Bujanda et al., 2019; Silbereisen et al., 2020). It remains in its latent proMMP-8 form throughout the induction and resolution of gingival inflammation. In patients with periodontitis, salivary MMP-8 is activated and fragmented, and studies have shown that aMMP-8, rather than total MMP-8 levels, correlates strongly with connective tissue destruction (Arias-Bujanda et al., 2019; Silbereisen et al., 2020). Monitoring aMMP-8 enzymatic levels and activation degree could aid in disease progression assessment (Arias-Bujanda et al., 2019). aMMP-8 activity and immunoreactivity are elevated in the gingival tissue, GCF, saliva, and mouth rinse of patients with periodontitis, making them useful for chair-side/point-of-care (PoC) lateral flow immunoassays (Gupta et al., 2023). Alassiri et al. found higher diagnostic sensitivity (76%–90%) and specificity (96%) for aMMP-8 PoC/chair-side tests, with an odds ratio of >72 (Arias-Bujanda et al., 2020).

#### 2.3.2 NE

NE is a serine protease stored in azurophilic granules and is released during bacterial phagocytosis or cell lysis, contributing to host defense by degrading pathogenic bacteria and extracellular matrix proteins (Kayar et al., 2020). This release leads to local tissue damage and regulation of inflammatory and repair processes (Kayar et al., 2021). NE can degrade connective tissue materials, alter cellular functions, and break down serum proteins, exerting potent pro-inflammatory effects (Kayar et al., 2021; Sriwattanapong et al., 2021; Tseng et al., 2022). In periodontitis, neutrophils migrate to inflamed sites and release excessive NE, leading to connective tissue degradation, exacerbation of inflammation, and periodontal tissue loss. Prolonged chronic inflammation results in the loss of periodontal ligament support and alveolar bone, potentially leading to tooth loss (Aral et al., 2020; Tseng et al., 2022). Studies using murine models of periodontitis have shown that NE activity is linked to periodontal bone loss, which can be mitigated using NE inhibitors (Hiyoshi et al., 2022). In human gingivitis, NE levels in the GCF increase, correlating with clinical attachment loss in patients with periodontitis (Hiyoshi et al., 2022). NE levels were significantly higher in patients with aggressive periodontal disease compared to healthy controls (Tseng et al., 2022). NE, the most concentrated proteinase in the GCF during inflammation (Kayar et al., 2020), is linked to the pathogenesis and progression of periodontal disease (Tseng et al., 2022). Cleaves cell adhesion molecules, disrupts gingival epithelial barrier and aggravates periodontitis. Periodontal pathogens such as *T.d* and *P. g* can stimulate NE release and evade host proteases (Hiyoshi et al., 2022). NE modulated the expression of cytokines, chemokines, and growth factors during infection (Sriwattanapong et al., 2021). It upregulates placental growth factors and contributes to the inflammatory cascade in periodontal disease. NE activates protease-activated receptors (PAR)-1, PAR-2, and PAR-4, promoting the secretion of IL-1β, TNF-α, and IL-6 by oral epithelial cells (Tseng et al., 2022). Due to its role in extracellular matrix degradation and tissue disruption, NE is a potential diagnostic marker for periodontal disease (Kayar et al., 2020; Kayar et al., 2021). GCF NE activity is a reliable indicator for diagnosing periodontal disease and alveolar bone loss, with a sensitivity and specificity of 84% and 66%, respectively, in one study, and 77% and 61%, respectively, in another (Kayar et al., 2020; Kayar et al., 2021). A recent meta-analysis by Arias-Bujanda et al. found NE to be the second most researched GCF biomarker, with median sensitivity and specificity of 75% and 81%, respectively (Arias-Bujanda et al., 2019).

### 2.4 Other enzymes associated with periodontitis

Many enzymes associated with periodontitis should be studied further. Arias‐Bujanda highlighted that the salivary biomarker Hb exhibits similar sensitivity (72%) to MMP8 and IL6 for diagnosing periodontitis, with even better specificity (75% compared to 70.5% and 73%, respectively) (Arias-Bujanda et al., 2020). Salivary alkaline phosphatase (ALP) levels are directly related to periodontal inflammation and tissue destruction, making ALP a strong biomarker for dental inflammation and bone regeneration (Tirachaimongkol et al., 2016; Kim et al., 2023). Osteoclast activation in periodontitis hinges on the balance between receptor activators of NF-κB (RANK), RANK ligand (RANKL), and osteoprotegerin (OPG). RANKL, a pro-osteoclastogenic protein, is elevated in the GCF of patients with chronic and aggressive periodontitis (stages II, III, and IV), whereas OPG, an anti-osteoclastogenic protein, counteracts these effects. Higher RANKL/OPG ratios in the GCF further support the increased osteoclast activity in periodontitis (stages II, III, and IV) (Caldeira et al., 2021).

### 2.5 Multiple biomarkers to diagnose periodontitis

Biomarkers are crucial for early diagnosis of periodontitis. Given the complexity of the disease, the use of multiple biomarkers may be necessary to account for the different inflammatory and tissue destruction components (Blanco-Pintos et al., 2023). However, many studies have focused on discovering new biomarkers rather than identifying effective biomarker combinations (Blanco-Pintos et al., 2023). Combining multiple biomarkers can offer a more precise evaluation of a patient’s periodontal condition, ideally using the fewest biomarkers necessary to achieve high diagnostic accuracy (Kc et al., 2020). Among extensively studied salivary biomarkers, the combination of MMP-8 and IL-6 has shown the highest diagnostic accuracy for periodontitis (Arias-Bujanda et al., 2020; Kc et al., 2020). A recent meta-analysis indicated that early periodontitis diagnosis accuracy significantly increases when detecting IL-1β alongside other biomarkers in saliva. Specifically, combinations such as MMP-8 with IL-1β, IL-1β with IL-6, or MMP-8 with IL-6 correctly identified 78%–81% of patients with periodontitis and excluded 85%–88% of non-periodontitis participants (Blanco-Pintos et al., 2023; Cennamo et al., 2024). MMP-8 combined with IL-1β can distinguish between periodontal health and periodontitis, even in patients with systemic inflammatory diseases such as type 2 diabetes mellitus (Cennamo et al., 2024). Studies have shown that oral rinse samples from periodontitis patients contain substantially higher levels of MMP-8 and TPA than those from controls (Katsiki et al., 2021). MMP-8 combined with chitinase accurately classified participants with 87.2% accuracy, distinguishing periodontitis from periodontal health/gingivitis (Katsiki et al., 2021). In saliva, MMP-8 combined with IL-1β, and IL-6, are excellent for detecting periodontitis and identifying non-periodontitis (Blanco-Pintos et al., 2023). The combination of MMP-8, IL-1β, IL-6, and MIP-1α has shown promise in distinguishing between gingivitis and periodontitis, although based on a smaller study sample (Arias-Bujanda et al., 2020; Kc et al., 2020). MMP-8, MMP-9, IL-1β, IL-6, and Hb have been effective salivary biomarkers for detecting periodontitis in systemically healthy individuals, while MMP-9 and IL-1β also perform well in identifying non-periodontitis conditions. Other biomolecules such as cysteine, MIP-1α, and nitric oxide and its metabolites have shown promise as salivary biomarkers, but further research is needed to validate their diagnostic potential (Arias-Bujanda et al., 2020). Blanco-Pintos et al. evaluated the diagnostic accuracy of various biomarker combinations in GCF and saliva. The six combinations of salivary biomarkers with the highest diagnostic accuracy show sensitivity and specificity as follows: MMP-8 with IL-6 (86.2%/80.5%), IL-1β with IL-6 (83.0%/83.7%), MMP-8 with IL-1β (82.7%/80.8%), MMP-8 with MIP-1α (71.0%/75.6%), MMP-8, IL-1β, and IL-1β(81.8%/84.3%), and MMP-8, IL-1β, IL-6, and MIP-1α(76.6%/79.7%). Tomás et al. (2017) demonstrated great sensitivity and specificity values (>90%) for the combination of pro-inflammatory and anti-inflammatory cytokines. Two-biomarker combinations of oral fluids have shown high diagnostic accuracy for periodontitis, with little improvement when additional biomarkers were added (Arias-Bujanda et al., 2020). However, the low levels of biomarkers in the GCF may limit their clinical applicability. Recently, high-throughput proteomics techniques have been used to identify new biomarkers that may improve the diagnosis of periodontitis. The biomarker combinations with the greatest diagnostic accuracy comprised four protein groups made up of 10 biomarkers, along with a five-protein combination (MMP-13, IL-1β, IL-8, OA, and OPG), all exhibiting sensitivity and specificity above 95% (Huang et al., 2020; Rizal et al., 2020). A more comprehensive evaluation of additional combinations of periodontitis biomarkers, beyond MMP-8 with IL-1β and IL-6, is warranted, along with the development of more sensitive biomarker assays.

## 3 NPs

**Figure 2:**
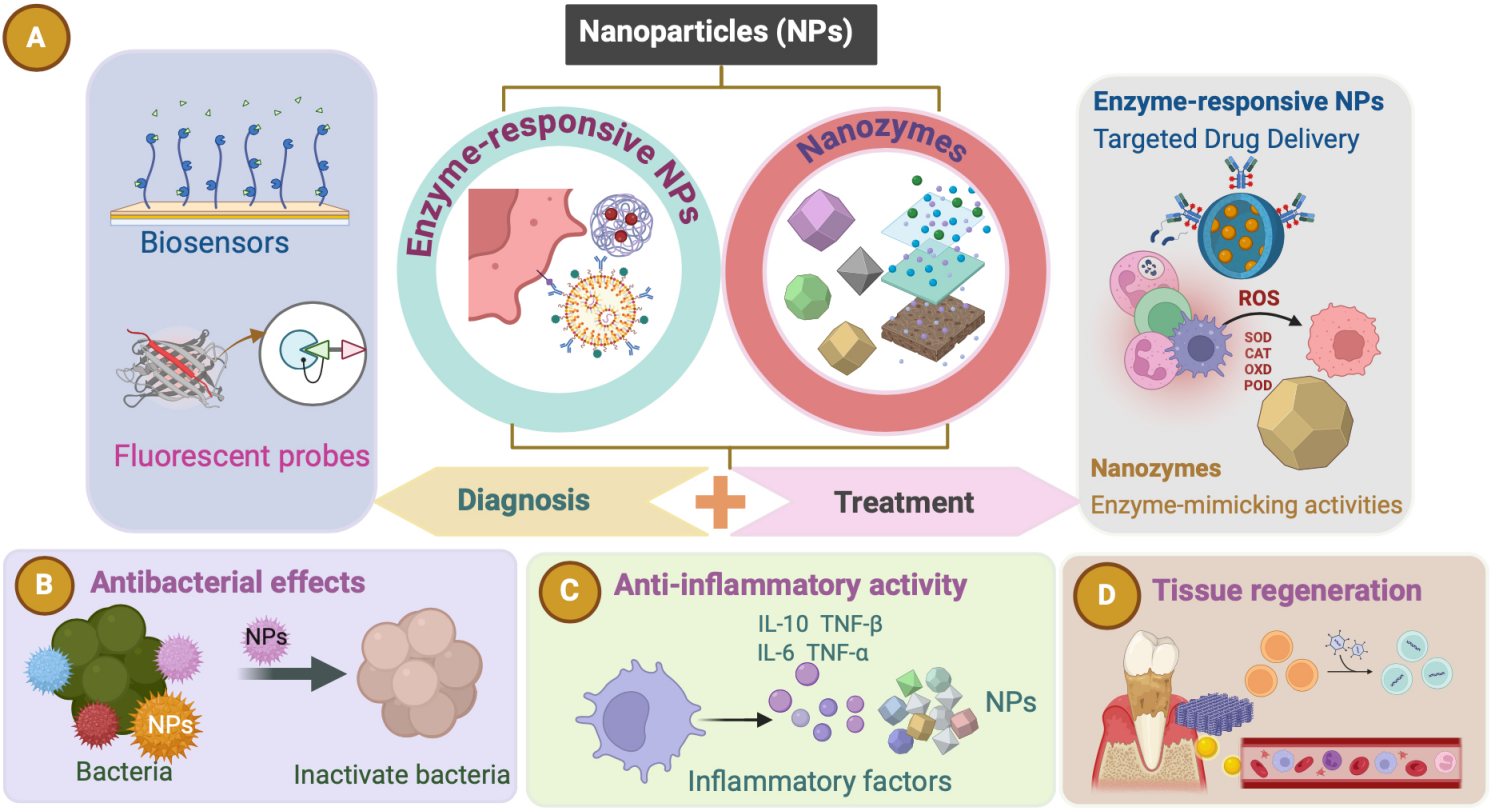
Nanoparticles (NPs). **(A)** Enzyme-responsive NPs and nanozymes (NZs) hold significant potential for advancing both diagnostic and therapeutic applications in periodontitis. These NPs can be used as biosensors and fluorescent probes for diagnosing periodontitis. Enzyme-responsive NPs and NZs both exert therapeutic effects in periodontitis. **(B–D)** Various effects (such as antibacterial effects, anti-inflammatory activity, and tissue regeneration) of the NPs as a therapeutic approach for periodontitis.

Enzymes associated with periodontal disease not only indicate the progression of periodontitis but also offer therapeutic potential through their regulation. Recent advances in NPs present new opportunities to modulate enzyme structures and catalytic activities (Lin et al., 2016), making them promising tools for the detection and treatment of periodontitis (Zhang S. et al., 2024) (**Figure 2**). NPs are valued for their ease of preparation, high surface-area-to-mass ratios, customizable surfaces, and unique electronic, optical, magnetic, and catalytic properties (De et al., 2011; Li et al., 2014). By integrating NPs with antimicrobial agents, antioxidants, or growth factors, multifunctional composites can be engineered to enhance therapeutic outcomes. These composites are designed to specifically target and inhibit key enzymes involved in periodontitis progression, such as gingipains and MMPs. NP-based sensors offer ultrasensitive detection of clinically relevant biomolecules, which is crucial for early disease diagnosis (de la Rica et al., 2012). For applications such as biosensors and controlled drug release, tailoring the surface chemistry of NPs is essential to ensure that their physicochemical properties align with their intended functions (de la Rica et al., 2012). Research efforts have increasingly focused on hybrid nanocomposites that combine the recognition and catalytic properties of enzymes with the electronic features of nanomaterials (Lin et al., 2016). This integration creates electrically active biomaterials, thereby introducing new dimensions into nanobioelectronics (Lin et al., 2016). Enzymes coupled with NPs can retain their natural catalytic abilities while enhancing the properties of the NPs (Lin et al., 2016). Combined with the unique characteristics of nanomaterials, enzyme-responsive NPs can be engineered to perform highly specific and efficient functions in response to particular stimuli (de la Rica et al., 2012). In oral therapy, NPs can be categorized by composition and structure into three main types: inorganic, organic, and inorganic–organic hybrids (Carrouel et al., 2020; Higino and França, 2022; Chen et al., 2023). By integrating enzymatic functions, NPs offer promising strategies for enhancing diagnostic and therapeutic precision while addressing delivery and toxicity challenges. We focuses on two types: enzyme-responsive NPs and nanozymes. Enzyme-responsive NPs activate or release therapeutic agents in response to disease-specific enzymes. Nanozymes, with intrinsic enzyme-mimicking activity, can catalyze reactions like peroxidase(POD) or CAT, aiding in the degradation of harmful biomolecules and modulation of oxidative stress, particularly in the management of periodontitis. These tailored NPs have significant potential for advancing both diagnostic and therapeutic applications in periodontitis (**Figure 2**).

### 3.1 Enzyme-responsive NPs

**Figure 3:**
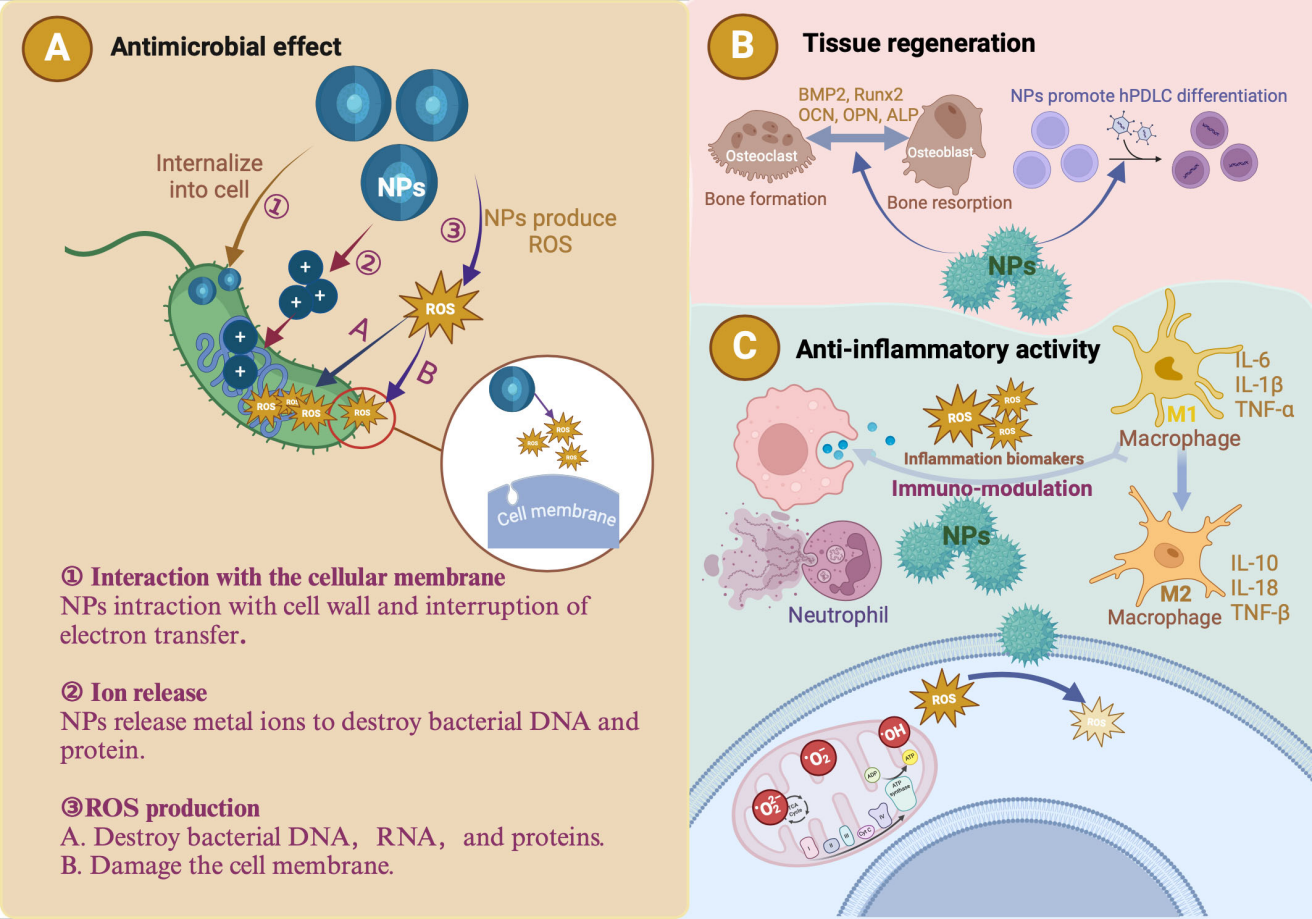
The antibacterial, anti-inflammatory and tissue regeneration mechanisms of nanoparticles (NPs). **(A)** The antibacterial effects of metallic NPs are attributed to three primary mechanisms: ROS production, ion release, and interaction with the cellular membrane. ROS generated by these NPs degrade essential cellular components (DNA, RNA, and proteins), effectively killing periodontal pathogens. Due to their small size, metallic NPs can penetrate the peptidoglycan layer and damage bacterial cells. The metal ions released by NPs are toxic to bacterial DNA and proteins. **(B)** Introduction of metallic NPs results in a microenvironment characterized by low levels of inflammatory cytokines and high levels of reparative cytokines, such as bone morphogenetic protein-2 (BMP-2), runt-related transcription factor 2 (Runx2), osteocalcin (OCN), osteopontin (OPN), and alkaline phosphatase (ALP). This favorable environment promotes human periodontal ligament cell (hPDLC) differentiation and periodontal tissue regeneration and halts the progression of periodontitis. **(C)** NPs exhibit antioxidant properties that help regulate ROS. Metallic NPs reduce inflammation by regulating cytokines and macrophage polarization.

NPs significantly enhance the effectiveness of bioactive molecules in periodontal therapy by improving penetration, drug release rate, and controlled delivery, all vital for tissue regeneration (Yang et al., 2020). Advancements in nanotechnology enable the targeted delivery of anti-inflammatory biomolecules and medications to specific locations within the oral cavity, such as periodontal pathogens, inflammatory cells, and surrounding tissues. Targeted delivery reduces the need for invasive procedures, minimizes side effects, and enhances treatment efficacy (Aral et al., 2020). Enzyme-responsive NPs exert their effects through multiple mechanisms. First, NPs may be composed of materials that are sensitive to enzymatic transformation, either because they contain chemical structures recognized by a biocatalyst, or because they can be modified by the products of enzymatic reactions (de la Rica et al., 2012). In addition, NP surfaces can be modified with molecules that undergo physical changes upon enzymatic transformation, allowing multimodal probes to perform various functions (de la Rica et al., 2012). Third, drug loading into nanocarriers can be achieved through methods such as covalent attachment, physical encapsulation within self-assembled systems, crosslinked matrices, or caged porous structures (Kapalatiya et al., 2022). These carriers are designed to target specific cells by exposing them to targeting ligands to ensure efficient internalization (Kapalatiya et al., 2022). A critical step in the fabrication of enzyme-responsive NPs is the use of robust immobilization techniques for surface modification along with the design of ligands that transduce enzymatic activity into physical changes in the NP solution (de la Rica et al., 2012). The fundamental principle underlying enzyme-responsive nanocarriers is their ability to undergo chemical and physical transformations upon exposure to enzymes, triggering drug release at the desired site. These bioactive carriers are classified based on their inclusion techniques. For example, when bioactive molecules are entrapped within a nanocarrier, drug release is typically triggered by structural changes in the carrier, such as the cleavage of charged functional groups, shell degradation, or splitting of a linker to release the bioactive molecule (Kapalatiya et al., 2022). In summary, NPs can exert good antibacterial and anti-inflammatory effects through the above methods, and we introduce their antibacterial, anti-inflammatory, and tissue regeneration-promoting mechanism (**Figure 3**).

#### 3.1.1 The antibacterial mechanism of NPs

Metals such as gold (Au), silver (Ag), titanium (Ti), copper (Cu), magnesium (Mg), zinc (Zn), and bismuth effectively inhibit bacterial growth and show significant potential in periodontitis treatment. Platforms using metal NPs ranging from 0.2 to 100 nm in size have been developed as antibacterial agents (Maleki Dizaj et al., 2015; Rudramurthy et al., 2016; Nasiri et al., 2023). NPs with antimicrobial properties include Au, Ag, Ag_2_O, titanium dioxide (TiO2), copper oxide (CuO), and zinc oxide (ZnO) (Priyadarsini et al., 2018). The antibacterial effects of metallic NPs can be attributed to three primary mechanisms: ROS production, ion release, and interactions with the cellular membrane (Kırmusaoğlu, 2018)(**Figure 3A**). ROS generated by these NPs, such as superoxide anions and hydroxyl radicals (-OH), degrade essential cellular components (DNA, RNA, and proteins) and effectively kill periodontal pathogens (Priyadarsini et al., 2018; Agarwal et al., 2019; Nasiri et al., 2023). Owing to their small size, metallic NPs can penetrate the peptidoglycan layer and damage bacterial cells (Safarov et al., 2019). The metal ions released by NPs can damage bacterial DNA and proteins (Agarwal et al., 2019; Nasiri et al., 2023). Negatively charged bacterial cell surfaces will absorb Cu^2+^, Ag+, and Zn^2+^ released by metallic NPs (Safarov et al., 2019). These ions penetrate the cells and react with sulfhydryl groups on microbial proteins, inhibiting their ability to synthesize proteins or nucleic acids (Agarwal et al., 2019; Nasiri et al., 2023). The effectiveness of NPs in eradicating bacteria depends on the particle size and surface charge. Smaller particle sizes and higher surface-to-volume ratios confer superior antimicrobial efficacy without compromising the mechanical properties of the material, making NPs highly effective in combating bacterial infections in periodontal disease (Agarwal et al., 2019; Nasiri et al., 2023). Periodontitis is primarily driven by pathogenic bacteria organized in biofilms composed of extracellular polymeric substances and microbial flora (Carrouel et al., 2020; Nasiri et al., 2023). These biofilms play a critical role in disease progression, making bacteria within them 1,000 times more resistant to various antibiotic treatments than planktonic bacteria, including penicillin, aminoglycosides, and quinolones (Carrouel et al., 2020; Gao et al., 2023; Nasiri et al., 2023). Furthermore, biofilms enable bacteria to evade the immune system by producing superantigens (Carrouel et al., 2020). Metals, metal oxides, and other NPs have emerged as promising alternatives to combat bacterial infections due to their enhanced antibacterial activity at the nanometer scale. NPs facilitate interaction and penetration of bacterial cells (Carrouel et al., 2020). NP-based photodynamic therapy (PDT) and photothermal therapy (PTT) have shown increasing potential in treating bacterial infections. PDT uses light to produce ROS that destroy bacterial molecules, whereas PTT transforms light into heat, causing bacterial destruction. Combining PDT and PTT enhances efficacy owing to the synergistic effects of ROS and heat (Gao et al., 2023).

#### 3.1.2 The anti-inflammatory mechanism of NPs

The inflammatory response and cell behavior are influenced by material properties such as surface chemistry, topography, microgrooved design, wettability, and the presence of biologically active proteins. These characteristics are crucial for determining the anti-inflammatory efficacy of NPs (Agarwal et al., 2019; Ghinassi et al., 2020). Metal and metal oxide NPs, including Au, Ag, ZnO, TiO_2_, CuO, and magnesium oxide, exhibit significant anti-inflammatory properties, making them effective in treating periodontal diseases by targeting both gram-positive and gram-negative bacteria (Agarwal et al., 2019). For NPs to exert anti-inflammatory effects, they must enter cells via ion channels or pores, and their size and concentration influence cellular uptake (Simko et al., 2011). Higher concentrations of small NPs are typically endocytosed, whereas macrophages and neutrophils undergo phagocytosis and macropinocytosis (Mahmoudi et al., 2011; Kuhn et al., 2014; Muñoz et al., 2016; Carrouel et al., 2020) (**Figure 3C**). Gold nanoparticles (GNSs) reduce ROS production, decrease lipopolysaccharide-induced cytokine production (IL-1β, IL-17, TNF-α), and modulate mitogen-activated protein kinase (MAPK) and phosphatidylinositol 3-kinase pathways (Agarwal et al., 2019). Silver nanoparticles (AgNPs) decrease levels of vascular endothelial growth factor (VEGF), reduce hypoxia-inducible factor 1α (HIF-1α) expression, prevent mucin (mucus glycoproteins) hypersecretion, suppress pro-inflammatory cytokines (IL-12, TNF-α), and decrease cyclooxygenase-2 (COX-2) gene expression (Agarwal et al., 2019). ZnO NPs block the production of pro-inflammatory cytokines (IL-1β, IL-18), inhibit mast cell proliferation by upregulating p53 and reducing thymic stromal lymphopoietin levels, and suppress lipopolysaccharide-induced COX-2 and inducible nitric oxide synthase expression (Agarwal et al., 2019). TiO2 NPs reduced platelet numbers and increased thrombin–antithrombin levels (Agarwal et al., 2019). These NPs, through modulating the expression of enzymes or genes, offer promising anti-inflammatory strategies for periodontal disease treatment.

#### 3.1.3 The tissue regeneration promotion mechanism of NPs

In addition to reducing inflammation, periodontal treatment aims to control infection and rebuild tissues such as the cementum, periodontal ligaments, and bone (Zhu et al., 2023). Kiarashi et al. demonstrated 45 nm AuNPs reduce inflammation and improve the periodontal ligament environment by regulating cytokines and macrophage polarization. This results in a microenvironment characterized by low levels of inflammatory cytokines and high levels of reparative cytokines such as bone morphogenetic protein-2. This favorable environment promotes the differentiation of human periodontal ligament cells and periodontal tissue regeneration and halts the progression of periodontitis (**Figure 3B**) (Kiarashi et al., 2024). Graphene, known for its mechanical properties, antibacterial activity, high surface area, and biocompatibility, promotes stem cell differentiation. Graphene oxide (GO) and reduced graphene oxide (rGO) are promising materials for tissue engineering that enhance the proliferation of periodontal ligament stem cells (PDLSCs), which are available for regenerative therapy (Liu C. et al., 2022). Polycaprolactone, used for three dimensional printing due to its favorable properties, can form bone–ligament–cementum complexes *in vivo (* Roato et al., 2022). Plant-derived exosome-like nanoparticles (PELNs), which are natural nanocarriers rich in lipids, proteins, RNAs, and other active molecules, can enter mammalian cells and regulate cellular activity. Literature reports indicate the great potential of PELNs in regulating immune function, inflammation, microbiome balance, and tissue regeneration (Zhang Z. et al., 2022). In conclusion, NPs offer significant advantages in periodontal tissue regeneration. Their unique properties, such as high surface-area-to-volume ratio and tunable surface chemistry, enable the development of advanced therapies to enhance tissue repair and overcome traditional treatment limitations.

### 3.2 Nanozymes (NZs)

**Figure 4:**
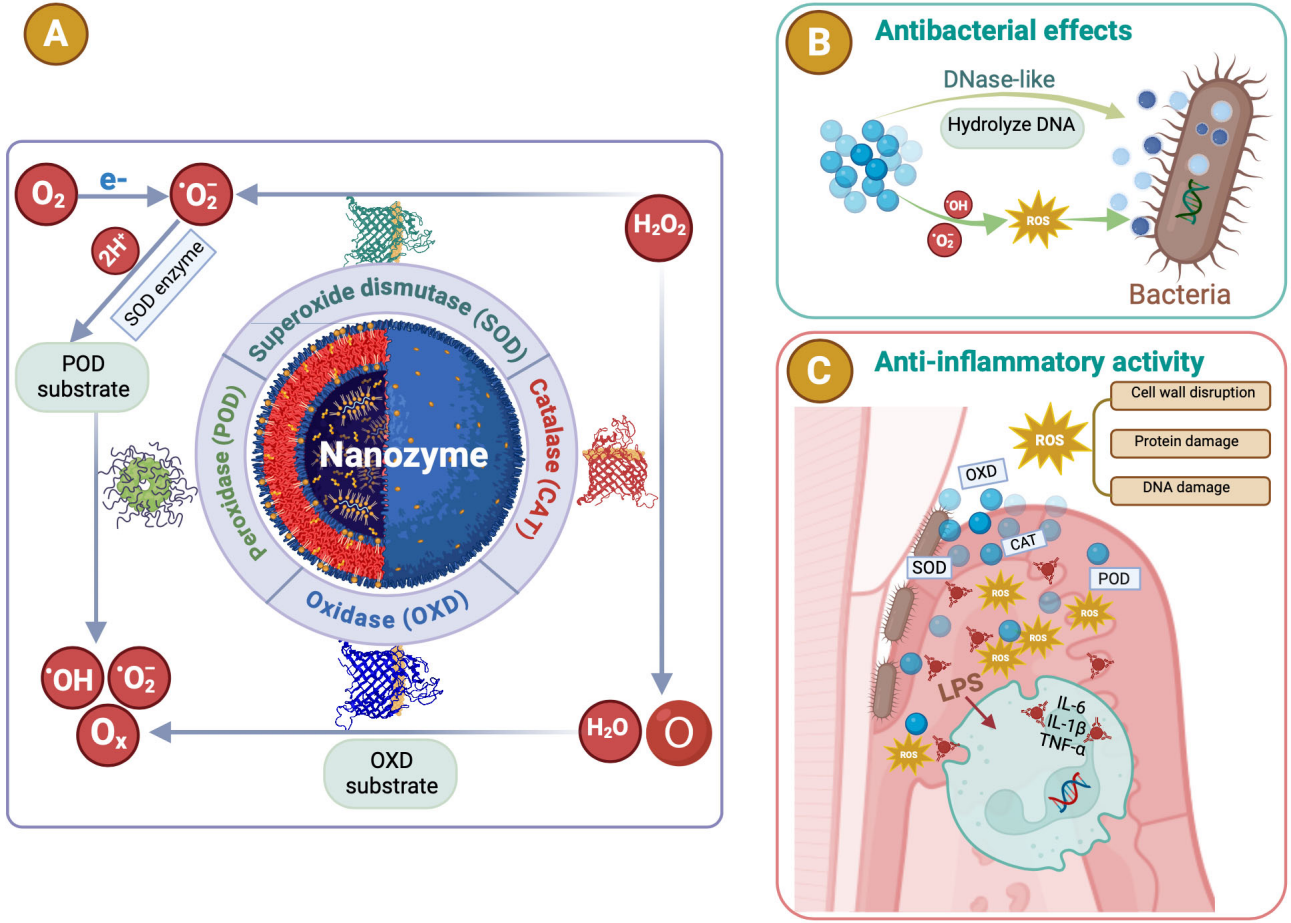
The antibacterial and anti-inflammatory mechanisms of nanozymes (NZs). **(A)** NZs are NPs with enzyme-mimicking properties. They mimic enzymes such as superoxide dismutase (SOD), catalase (CAT), oxidase (OXD), and peroxidase (POD). POD or oxidase mimics converting hydrogen peroxide (H_2_O_2_) or superoxide (O_2_) into ROS such as hydroxyl radicals (·OH) or singlet oxygen (^1^O_2_). **(B)** The antibacterial processes and properties of NZs. Catalytic activities of NZs efficiently eliminate bacterial biofilms, showing broad-spectrum antimicrobial activity with minimal biotoxicity. **(C)** Various NZs exhibit antioxidant properties that help regulate ROS. NZs suppressed inflammatory responses by eliminating ROS, while also lowering pro-inflammatory cytokines and boosting anti-inflammatory cytokines.

Enzymes are crucial in treating periodontitis by degrading bacterial biofilms and alleviating inflammatory responses, thereby promoting periodontal tissue healing and repair. Proteases such as trypsin and Bacillus proteases (AprBp and GseBp) degrade the EPS matrix and adhesion proteins in biofilms, thereby improving the effectiveness of therapeutic agents and reducing bacterial virulence. These attributes make proteases a valuable choice for antibacterial and anti-biofilm applications (Gao et al., 2023). However, natural enzymes face significant drawbacks, including poor stability under extreme conditions, high production costs, lengthy separation processes, and storage challenges (Liang and Yan, 2019; Chen et al., 2021; Zhu et al., 2023; Hosseini Hooshiar et al., 2024). Recently, NPs with enzymelike properties have emerged as promising alternatives. NZs are NPs with enzyme-mimicking properties (Wei and Wang, 2013), including metals (e.g., Au, Ag, platinum (Pt), Pd, Ir, and Au@Pt), metal compounds (e.g., Fe_3_O_4_, cerium oxide (CeO_2_), MnO_2_, CuS, and MnSe), non-metals (e.g., SWNTs, C-dots, and fullerene), and non-metal compounds (e.g., g-C3N4 and GO) (Yang et al., 2021). They mimic enzymes such as SOD, CAT, oxidase (OXD), and POD (Zhang Q. et al., 2022; Hosseini Hooshiar et al., 2024) (**Figure 4A**), and can be classified into two categories: (1) oxidoreductase-like nanozymes, such as OXD and POD, and (2) hydrolase-like nanozymes, including nuclease and protease (Huang et al., 2019). Like natural enzymes, NZ activity is influenced by factors such as pH, temperature, and metal ions (Huang et al., 2019). By combining the characteristics of chemicals and biocatalysts, NZs can be used in biosensing, environmental remediation, disease treatment, antibacterial applications, and cytoprotection (Huang et al., 2019). NZs can be categorized into two types: (1) nanomaterial hybrid enzymes, in which enzymes or catalytic groups are modified on nanomaterials to enhance their stability and durability, and (2) nanomaterials with inherent enzyme-like catalytic properties that can mimic natural enzyme reactions (Zhou et al., 2017). Natural enzymes are proteins with high catalytic efficiency (Wu et al., 2019) and are composed of amino acids, metal atoms, and cofactors (Gao et al., 2020). Their active sites, such as heme groups coordinated with metals such as Fe or Cu, are key to their function (Yang et al., 2021). When nanomaterials are reduced to the nanoscale, they acquire distinct physicochemical properties compared to their bulk counterparts. The high energy and instability of the surface atoms, caused by undercoordination and suspended bonds (She, 2009), confer NZs with unique chemical reactivity. This is particularly evident in single-atom NZs, which display superior catalytic performances owing to their enzyme-like structures. Moreover, NZs can catalyze bioorthogonal reactions that natural enzymes cannot, likely because of their multivalent elements, multiple active sites, and diverse coordination structures (Dong et al., 2019). In summary, the nanoscale dimensions of NZs endow them with novel catalytic activity. Their catalytic efficiency is influenced by size, morphology, and external stimuli such as light, sound, and heat (Gao et al., 2007; Yang et al., 2021). Generally, smaller particle sizes enhance the catalytic activity (Gao et al., 2007) owing to an increased specific surface area, higher surface atom count, and elevated surface energy (Yang et al., 2021). The crystal field environment and binding energy of surface atoms differ from those of internal atoms, leading to the presence of dangling and unsaturated bonds (She, 2009; Peng et al., 2021). These highly reactive surface atoms contributed to the pronounced chemical and catalytic activities observed in the NZs (Yang et al., 2021). Extensive research has focused on these nanozymes (NZs) with enzyme-like activities (Ren et al., 2022), which are more affordable, stable, and easier to produce than natural enzymes. These properties make NZs highly promising for application, particularly in microbial infection treatment (Zhang Q. et al., 2022; Hosseini Hooshiar et al., 2024). In dentistry, NZs have shown great potential for antibacterial applications, particularly against resistant bacteria. Enzyme-like activities combined with PTT and PDT boost the antibacterial efficacy. Significant efforts have been made to develop antibacterial NZs such as carbon dots (CDs), carbon nanotubes, graphene oxide, and metal-organic frameworks (MOFs), which effectively destroy pathogenic biofilms responsible for oral diseases such as periodontitis (Dragoš and Kovács Á, 2017). In summary, NZs exhibit broad-spectrum antibacterial activity, lower drug resistance, and remarkable stability, making them promising for periodontics. Their application in maintaining periodontal health highlights their potential as novel antibacterial agents in dentistry (Su et al., 2022; Hosseini Hooshiar et al., 2024; Shahrtash et al., 2024).

#### 3.2.1 The antibacterial mechanism of NZs

NZs are garnering significant attention owing to their affordability, excellent stability, scalability, and multifunctionality. Unlike conventional antibiotics, NZs are less likely to induce bacterial resistance (Attar et al., 2019), owing to their favorable membrane permeability and biocompatibility. Their catalytic activities effectively eliminate bacterial biofilms, and their unique physicochemical properties allow for additional functionalities that are not found in natural enzymes. By modulating the composition, size, and shape of the NZs, they can perform multiple functions (Hosseini Hooshiar et al., 2024). As highly effective nanoantibiotics, NZs bridge the gap between biology and nanotechnology by exhibiting broad-spectrum antimicrobial activity with minimal biotoxicity (Yang et al., 2020). NZs act through several mechanisms. First, POD or oxidase mimics convert H_2_O_2_ or O_2_ into ROS such as singlet oxygen (1O2) or -OH (Fang, 2011), leading to irreversible bacterial and biofilm destruction (**Figure 4B**). Second, NZs can transform prodrugs into active antibiotics, thereby enhancing their antibacterial effects. Third, the phosphatase-like activity of NZs can break down bacterial cell membranes, causing bacterial death (Kim and Kim, 2021; Hosseini Hooshiar et al., 2024). Among these, ROS generation has been the most extensively studied. ROS display rapid wide-ranging activity against bacteria and cancer cells without promoting drug resistance. They can also attack the DNA and lipids of dormant bacteria, including “superbugs” and persistent biofilms (Wang et al., 2022) (**Figure 4B**). Their remarkable properties have made them a focal point of research, marking significant advancements in the development of novel antibacterial agents.

#### 3.2.2 The anti-inflammatory mechanism of NZs

Excessive ROS levels can lead to uncontrolled inflammation, which is a key factor in the development of numerous diseases (Gao et al., 2017). Thus, NZs with ROS-scavenging capabilities offer a promising approach for mitigating inflammation and preventing related disorders (Munir et al., 2020). Various NZs exhibit antioxidant properties that help to regulate ROS (Yang et al., 2021)(**Figure 4C**). Singh et al. revealed that low-dose cerium NPs efficiently neutralize free radicals in human liver cells, preventing early stage apoptosis and DNA damage without activating the body’s natural antioxidant defenses (Singh and Singh, 2019). CeO_2_’s ROS scavenging abilities stem from its ability to mimic superoxide dismutase and catalase, which also proved effective in reducing inflammation when used to coat Ti surfaces for peri-implantitis prevention (Li et al., 2019). Additionally, CeO_2_ NPs grown on montmorillonite surfaces successfully suppress inflammatory responses by eliminating ROS such as superoxide (O_2_), H_2_O_2_, and -OH, while lowering pro-inflammatory cytokines and boosting anti-inflammatory cytokines (Zhao et al., 2020). Similarly, vanadium pentoxide nanowires demonstrated strong antioxidant effects by mimicking the enzyme glutathione peroxidase, which allows them to neutralize ROS under physiological conditions (Vernekar et al., 2014). NACu-Cys NZs with superoxide dismutase-like activity eliminate ROS and protect against oxidative damage (Dashtestani et al., 2018). Manganese oxide (Mn_3_O_4_) NPs, due to their dual oxidation states (Mn^2+^ and Mn^3+^), also displayed enzyme-mimicking properties such as superoxide dismutase and catalase activities, enabling them to effectively scavenge O_2_ and -OH (Yao et al., 2018). These NPs are more stable than natural enzymes and surpass the CeO_2_ NZs in their ability to eliminate ROS (Miao et al., 2020). Furthermore, Cao et al. introduced ultra-small polyethylene glycol-coated rhodium nanodots, which exhibited impressive scavenging abilities for reactive oxygen and nitrogen species (RONS) and photothermal activity. These nanodots reduced pro-inflammatory cytokine levels (**Figure 4C**), such as TNF-α and IL-6, through their multiple enzyme-like activities against RONS. This led to a significant reduction in inflammation in dextran sulfate sodium-induced colitis model, suggesting its potential for treating inflammatory bowel diseases (Yang et al., 2021).

## 4 NPs used in periodontitis diagnosis

**Figure 5:**
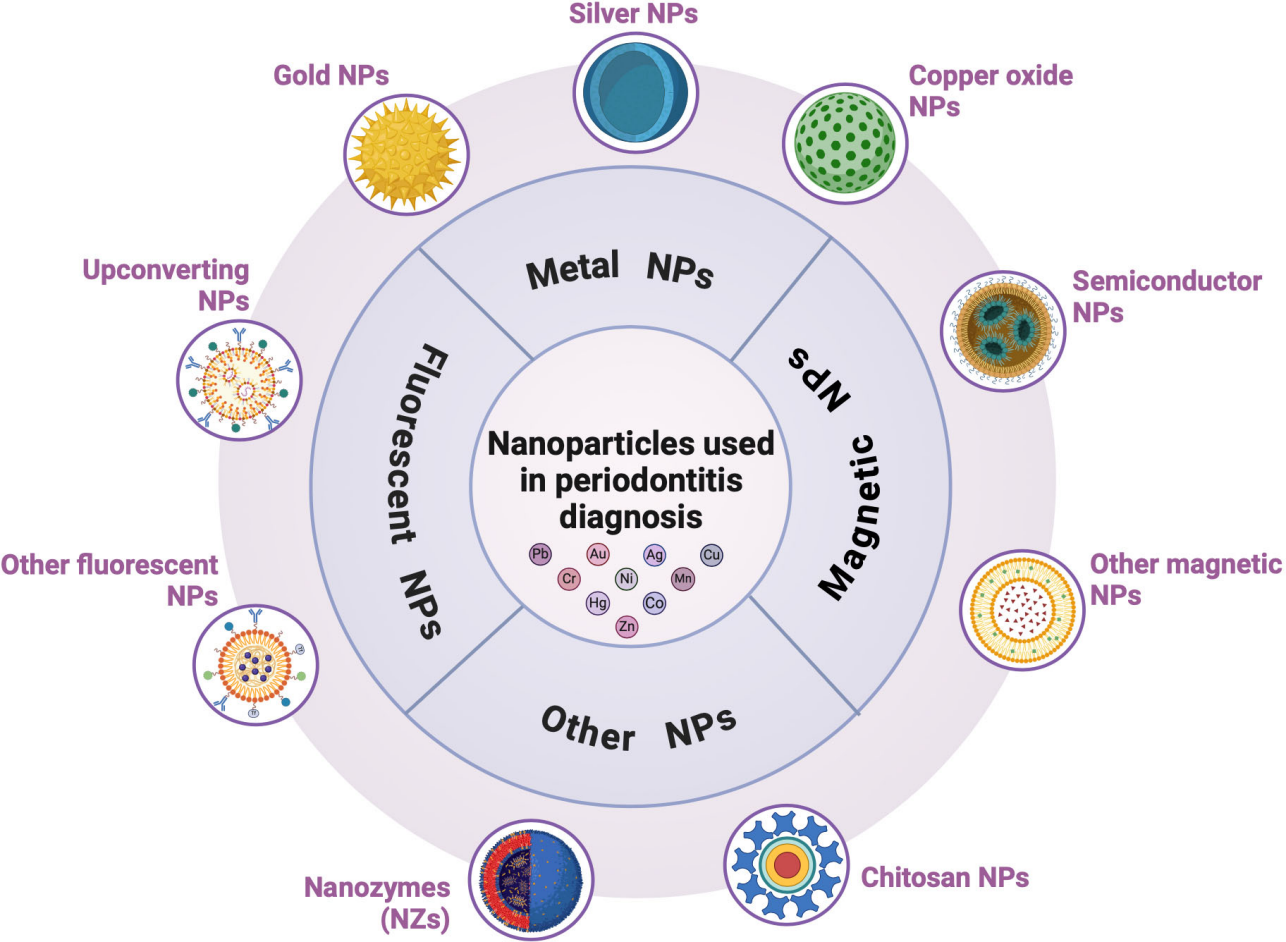
Nanoparticles (NPs) in periodontitis diagnosis. NP-based detection strategies are gaining popularity for the development of quick and efficient diagnostic assays. They include metal (gold, silver, and copper oxide), magnetic, and fluorescent NPs.

The accurate and timely diagnosis of periodontitis is essential for developing tailored and effective treatment regimens (Hooshiar et al., 2024). Early detection is crucial for chronic periodontitis to prevent severe and irreversible tooth damage as it progresses painlessly, and patients often do not seek dental treatment in the early stages. Traditional diagnostic methods, such as clinical inspection and X-rays, typically reveal tissue destruction and are ineffective for prevention or early diagnosis. Therefore, the non-invasive detection of predictive biomarkers in oral fluids is crucial (Tortolini et al., 2024). Accurate qualitative and quantitative detection of these enzyme biomarkers can improve diagnostic accuracy, identify current disease activity, predict disease progression, and monitor therapeutic intervention efficacy (Arias-Bujanda et al., 2019; Cennamo et al., 2024). Nanostructured materials have rapidly advanced bioassay methodologies, and NP-based detection strategies are gaining popularity for creating quick and efficient diagnostic assays. These platforms utilize the unique properties of NPs to identify periodontitis (Hooshiar et al., 2024) (**Figure 5**). Nanomaterials are categorized by shape and size (e.g., nanotubes, nanofibers, thin coatings, nanorods, and NPs), and composition (e.g., metal nanomaterials such as silver nanoparticles, CuO, TiO_2_, Mg, and iron compounds; non-metallic nanomaterials such as graphene, chitosan, hydroxyapatite NPs, bioactive glass, and mesoporous calcium silicate; semiconductor nanomaterials; and polymer nanomaterials). Various types of NPs, including metallic, magnetic, and fluorescent NPs, have been effectively utilized to detect infectious diseases. The unique electrical, magnetic, luminescent, and catalytic properties of NPs facilitate the rapid, sensitive, and efficient detection of microbial agents (Hooshiar et al., 2024). Point-of-care testing (POCT) revolutionizes medical diagnostics by enabling tests to be performed near the patient rather than in a laboratory. Biosensors and fluorescent probes are effective POCT tools for diagnosing periodontitis. Plasmonic NPs, such as metal NPs, carbon-based NPs, quantum dots (QDs), and nanozymes, are strong light absorbers and scatterers that make them ideal for biosensors. These NPs enhance the sensitivity and precision of labeling, surface-enhanced spectroscopy, and color-change sensors. Fluorescent NPs, including QDs, magnetic NPs, and metallic NPs (e.g., gold and silver NPs), serve as durable probes capable of labeling multiple biological targets and are effective for imaging, monitoring, and identifying a broad spectrum of infectious microorganisms (Hooshiar et al., 2024).

A biosensor is an analytical device that integrates a biologically active component with a physical transducer to generate a measurable signal that is directly related to the amount of chemical compounds in a sample. Biosensors are utilized in pharmaceutical studies, disease monitoring, and detecting pathogens, pollutants, and disease markers in physiological fluids (e.g., blood, saliva, GCF, urine, and sweat). Recent advancements have focused on the development of biosensors for real-time health surveillance, prevention, and treatment. With the rise of smart wearable devices, these technologies can predict diseases and further enhance their utility (Malik and Waheed, 2023; Hooshiar et al., 2024). Nanoscale biosensors are particularly suitable for diagnosing periodontal diseases. These sensors integrate transducers, including optical, piezoelectric, and electrochemical devices with biological detection components (e,g., enzymes, antibodies and nucleic acids). This close association enhances the precision and quantitative capabilities of biosensors in understanding biodegradation processes (Hooshiar et al., 2024).

### 4.1 Metal NPs used in periodontitis detection

#### 4.1.1 Gold NPs

Among various nanomaterials, GNSs are particularly notable in bioengineering and offer significant advancements in diagnostic tools and treatments for severe diseases. GNSs are recognized for their biocompatibility, stability, low toxicity, and ability to efficiently manipulate energy and matter at the nanoscale level. In diagnostics, GNSs can be engineered to bind to specific biomolecules, enabling highly sensitive and precise disease detection. They are particularly effective in the early diagnosis and prevention of periodontal disease, overcoming the challenge of collecting sufficient fluid for analysis. Their unique physicochemical properties, including size- and shape-dependent optical characteristics, surface chemistry, and biocompatibility, have positioned them at the forefront of early dental disease detection (Zhang S. et al., 2024).

The primary bacterium responsible for periodontitis, *P.g*, secretes harmful proteases known as gingipains. Detecting gingipain activity in the gingival fluid is promising for early diagnosis and treatment. A previous study developed a nanoplasmonic biosensor using GNPs to detect gingipain proteolytic activity. GNSs were permitted to self-assemble into sub-monolayers within multi-well plates coated with casein or IgG. Proteases in the buffer catalyze the proteolytic degradation of casein as a substrate, resulting in a blue shift of the localized surface plasmon resonance (LSPR) band by 1–2 nm, depending on the concentration and duration. A significant LSPR change was observed only in samples with gingipain activity. The sensor’s limit of detection (LOD) was < 0.1 μg/mL, significantly lower than gingipain concentrations found in patients with severe periodontitis. This biosensor can quickly detect protease activity, thereby facilitating chair-side diagnosis (Svärd et al., 2020). Verma et al. created an electrochemical immunosensor using a composite of GNP and rGO to detect the salivary biomarker interleukin-8 (IL-8) non-invasively, with rapid detection in 9 min and high sensitivity across a range of 500 fg/mL – 4 ng/mL (Verma et al., 2017; Zhang S. et al., 2024). The accurate identification of MMP-8 enzyme is crucial for assessing periodontal disease progression. Tortolini et al. described a novel voltammetric sensor for detecting salivary MMP-8 using a graphene screen-printed electrode enhanced with GNPs and MMP-8-specific antibodies. Graphene is of particular interest owing to its unique electronic properties. When combined with metallic NPs, the electrocatalytic activity of graphene was significantly enhanced, improving the electron transfer pathways. The sensor showed excellent performance with a linear range from 2.5 to 300 ng/mL, an LOD of 1.0 ± 0.1 ng/mL, and a sensitivity of 0.05 μA mL cm² ng^-^¹. This disposable and economical biosensor is highly attractive for detecting MMP-8 in human saliva (Tortolini et al., 2024).

#### 4.1.2 Silver NPs

AgNPs are important for the development of bioengineering materials. Their unique properties make them invaluable for applications in targeted drug delivery, imaging, and nanotherapeutics. Salivary biomarkers such as ALP and IL-1β are critical in diagnosing periodontal diseases (Kim et al., 2023). ALP, which is detectable in the saliva through secretion, GCF from tissue degradation, and bacterial cells, normally ranges between 25 and 100 U/L. Several strategies exist for ALP assays, including colorimetric, fluorometric, Raman scattering, capillary electrophoresis, and electrochemical biosensors (Hafez et al., 2021). One study developed a multicolorimetric ALP and IL-1β sensing platform based on the geometric modification of a silver nanoplate transducer. ALP dephosphorylates p-aminophenol phosphate (p-APP) to p-aminophenol (p-AP), altering the LSPR of silver nanoplates and changing their color from blue to yellow. This multicolor sensor detects ALP in the range of 0 – 25 U/L with a LOD of 0.0011 U/L and IL-1β from 0 to 250 pg/mL with a LOD of 0.066 pg/mL, significantly lower than the conventional monochromatic ELISA (LOD of 3.8 pg/mL) (Kim et al., 2023). Hafez’s multicolor biosensor modified gold nanobipyramids (AuNBPs) with Ag nanoshells. The nanostructures consisted of an AuNBP core surrounded by an Ag nanoshell. This bioassay leverages the selective catalytic activity of ALP to dephosphorylate the glucose phosphate substrate into glucose, which subsequently reduces Ag ions to form nanoshells on the surface of the AuNBPs. A binary surfactant mixture of cetyltrimethylammonium bromide and Tween 20 was used to achieve a maximal blue shift of the LSPR with a high-resolution multicolor response correlated with the ALP concentration. This surfactant mixture promotes the uniform growth of Ag on the AuNBP surface, resulting in distinctive color changes corresponding to the ALP concentration. The released glucose induces a blue shift in the LSPR from 740 to 550 nm, with visible color transitions from yellowish-brown to green, blue, purple, and red as the ALP concentration increases from 0 to 50 U/L. These spectral changes demonstrated high sensitivity for ALP detection, achieving a low detection limit of 0.085 U/L (Hafez et al., 2021). These advanced sensors significantly improve the detection and monitoring of ALP and IL-1β levels in saliva, aiding in the early diagnosis of periodontal diseases.

#### 4.1.3 Copper oxide NPs

Copper oxide nanoparticles (CuO NPs) have shown promise for biosensor development. One study developed a non-enzymatic, highly electrocatalytic H_2_O_2_ biosensor using an electrode composed of chitosan, black phosphorus nanosheets (BP NSs), and CuO NPs. The integration of CuO and BP provided outstanding electrocatalytic performance, featuring a low detection limit of 30 nM, high sensitivity of 138.00 μA mM^-^¹ cm^-^², excellent selectivity, reusability, and long-term stability. This sensor effectively measured H_2_O_2_ levels in saliva and GCF samples, enabling precise differentiation between periodontitis patients and healthy individuals. Furthermore, the device successfully detected H_2_O_2_ release from macrophages and gingival fibroblasts at the cellular level, highlighting its potential for the early diagnosis of periodontal disease (Wang K. et al., 2021).

### 4.2 Magnetic NPs used in periodontitis detection

Magnetic nanomaterials are ideal biosensors that target proteases due to their unique properties. Alhogail et al. developed a gold biosensing platform that immobilized a gingipain-specific peptide on magnetic nanobeads using a gold-thiol linkage, demonstrating high sensitivity and specificity for detecting *P. g* at concentrations as low as 49 cfu/mL within 30 s (Alhogail et al., 2018). Wignarajah et al. developed a portable diagnostic biosensor targeting the salivary biomarkers human NE and cathepsin-G. This biosensor uses targeted protease probes attached to magnetic beads and a gold sensor surface. Upon proteolysis, the fragmented magnetic beads were attracted to an external magnet, revealing the gold sensor surface, which was visible without instrumentation. The biosensor detected HNE and cathepsin-G in both saliva samples and liquid solution, with minimum detectable concentrations of 1 pg/mL for HNE and 100 fg/mL for cathepsin-G. The analysis of samples from patients with periodontitis and healthy control confirmed its effectiveness, making it promising for affordable point-of-care (POC) devices (Wignarajah et al., 2015).

### 4.3 Fluorescent NPs used in periodontitis detection

#### 4.3.1 Up-converting NPs

Up-converting nanoparticles (UCNPs) are luminescent nanomaterials that convert low-energy photons into high-energy emissions. They possess unique photophysical properties such as resistance to photobleaching, low background autofluorescence, high penetration depth, photostability, low toxicity, and long luminescent lifetime. These attributes make UCNPs highly suitable for applications in biosensing and imaging (Duan et al., 2018; Zhang et al., 2019).

Accurate detection of matrix metalloproteinase-2 (MMP-2) is crucial for the early diagnosis, prognosis, and treatment of oral diseases (Wang et al., 2016; Schmitt et al., 2019; Liu L. et al., 2022; Zhang S. et al., 2022). A fluorescence resonance energy transfer (FRET)-based ratiometric fluorescent nanoplatform (UCNPs@SiO2@Cy3-pep) was developed to detect MMP-2. This platform used SiO2-coated UCNPs modified with a cyanine 3 (Cy3) polypeptide containing an MMP-2-specific peptide substrate (PLGVR). In the FRET system, the UCNPs served as the energy donor and Cy3 as the energy acceptor. The green luminescence of UCNPs@SiO2 was quenched by Cy3, whereas the red luminescence remained unaffected. Upon cleavage of Cy3 at the PLGVR peptide by MMP-2, green luminescence was recovered, allowing the detection of MMP-2 activity. Both *in vitro* and *in vivo* experiments confirmed the effectiveness of the nanoplatform for monitoring MMP-2 levels, showing great potential for periodontitis diagnosis (Bi et al., 2023).

The rapid and multiplex detection of biomarkers in the GCF is vital for diagnosing periodontitis progression and severity. Scientists have developed a disk-like lateral flow immunoassay strip (LFIS) using green core–shell UCNPs (G-UCNPs) as luminescent probes. This strip simultaneously detects three periodontitis biomarkers: MMP-8, IL-1β, and TNF-α. The G-UCNP-LFIS platform demonstrated high sensitivity and specificity in spiked GCF, with detection limits of 5.455, 0.054, and 4.439 ng/mL in the standard solutions. It showed stable recovery rates in modified artificial saliva and high correlation with clinical methods (0.995 for MMP-8, 0.976 for IL-1β, and 0.977 for TNF-α). The entire detection process was completed within 30 min, making this tool highly promising for managing and treating periodontitis (He et al., 2021).

#### 4.3.2 Other fluorescent NPs used in periodontitis detection

Organic fluorescent nanoparticles also play a critical role in the development of fluorescent probes. A coumarin-based fluorescent probe (named ZPS-NO), was developed to detect nitric oxide (NO). Excessive NO can react with molecular oxygen to form highly oxidative compounds such as N_2_O_3_ or peroxynitrite causing significant cell damage and potentially leading to tumors, inflammation, and other diseases (Zhang Y. et al., 2024). In healthy individuals, salivary NO concentrations range from 10 to 20 μM, whereas patients with oral periodontitis exhibit levels from 50 to 90 μM (Zhang Y. et al., 2022). The ZPS-NO probe, consisting of coumarin and o-phenylenediamine connected via a double bond, functions as a “turn-on” fluorescent probe. The electron-donating property of o-phenylenediamine induces the photoinduced electron transfer (PET) effect, quenching fluorescence. When NO is present, the PET effect is inhibited, resulting in a strong fluorescence “turn-on” effect. The ZPS-NO probe has an ultra-low NO detection limit of 14.5 nM, with detection results statistically comparable to those obtained using a commercial kit, demonstrating good applicability for NO detection in real saliva samples (Zhang Y. et al., 2024). Numerous small molecules and NPs contrast agents have been developed for photoacoustic imaging, with a particular emphasis on activatable probes for molecular imaging. Moore et al. introduced a dual-modal imaging probe, C2A, for detecting gingipain proteases secreted by *P.g*. This probe utilizes the intramolecular dimerization of peptide-linked cyanine dyes to create fluorescent and photoacoustic off states. Upon cleavage by Arg-specific gingipain (RgpB), the probe showed a 5-fold enhancement in photoacoustic signals and over 100-fold increase in fluorescence, with detection limits of 1.1 nM RgpB and 4.4E4 CFU/mL bacteria. The evaluation of GCF samples from 14 participants with and six without periodontal disease showed a strong correlation with qPCR-based detection of *P.g.* (Pearson’s r = 0.71), particularly in cases of severe disease progression (Moore et al., 2022). Advancements in NPs have enhanced gingipain detection methods. The existing strategies include nanobody immunoassays (Alhogail et al., 2018), electrochemical biosensors, fluorogenic dipeptides (Kaman et al., 2012), peptide-functionalized magnetic nanobeads, and protein-functionalized GNSs. These methods have reported detection limits of 7.81E6 CFU/mL bacteria, 5E5 CFU/mL bacteria, 1E5 CFU/mL bacteria, 49 CFU/mL bacteria, and 4.3 nM Kgp (CFU/mL not reported) (Kaman et al., 2012; Alhogail et al., 2018).

NPs significantly advance the early diagnosis and dynamic monitoring of periodontitis by targeting enzymes (mentioned above). Their integration into biosensors and fluorescent probes enhances their sensitivity, specificity, and practicality, making them valuable tools for detecting early-stage periodontal disease. These advancements have provided timely and effective management of periodontitis, representing a promising development in the field of oral diagnostics.

## 5 NPs used in periodontitis treatment

### 5.1 Metal NPs used in periodontitis treatment

**Figure 6:**
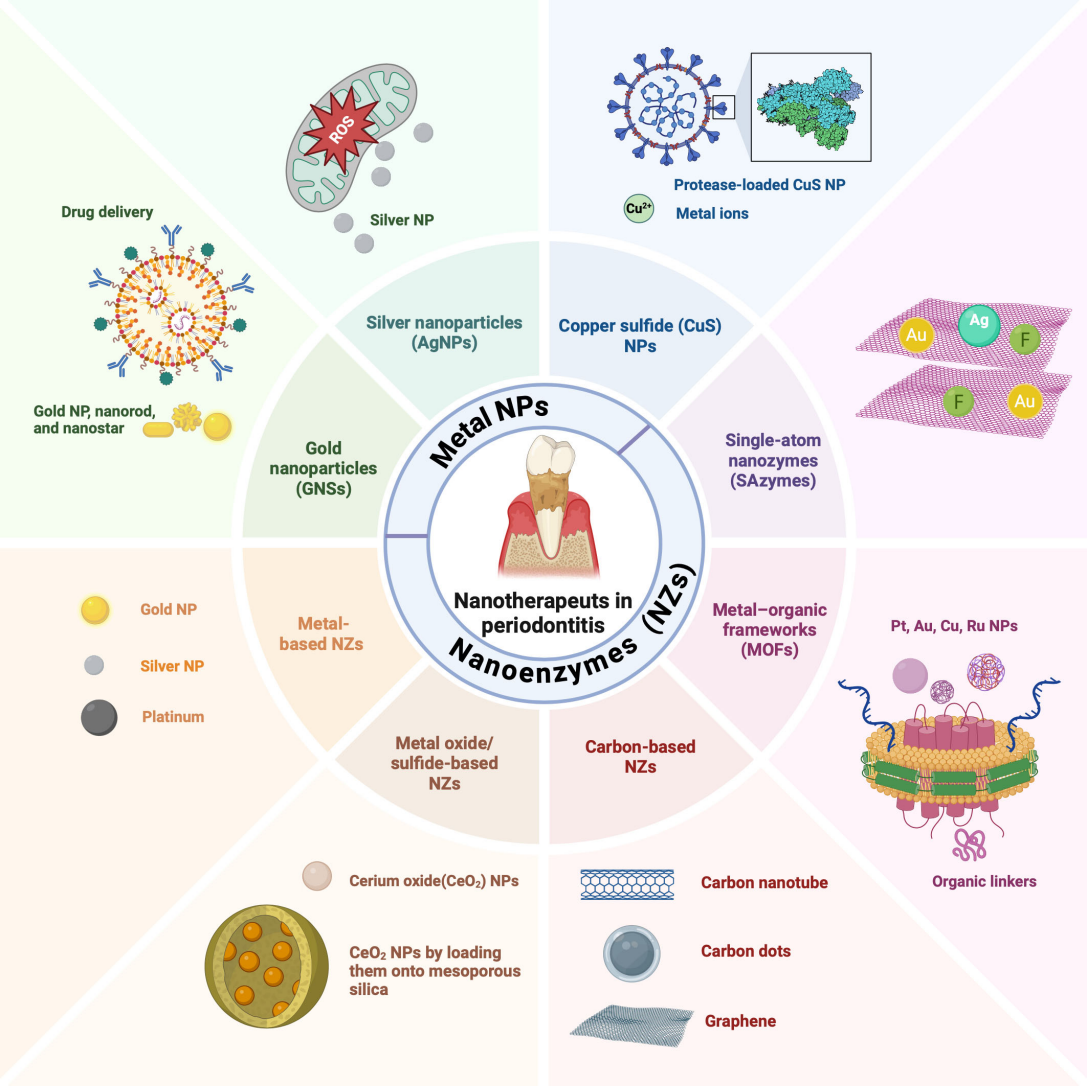
Nanoparticles (NPs) in periodontitis treatment. Metal NPs (gold, silver, copper oxide) and nanozymes (NZs) exhibit considerable promise in periodontal disease treatment. NZs are primarily composed of metal‐based, metal oxide/sulfide‐based, and carbon‐based NZs, as well as metal–organic frameworks (MOFs) and single-atom nanozymes (SAzymes).

Multifunctional metal NPs exhibit considerable promise in periodontal disease treatment due to their low toxicity, photothermal capabilities, immunotherapy potential, anti-inflammatory activity, antibacterial qualities, simple manufacturing, and low cost (Nasiri et al., 2023) (**Figure 6**). Their biocompatibility, prolonged shelf life, and high surface-area-to-volume ratios enhance their effectiveness as antibacterial agents. The rise of drug-resistant strains and growing microbial resistance to antibiotics has further increased scientific interest in these NPs. Their small size allows them to penetrate biofilm matrices, thereby enabling direct contact with bacterial cells and inhibiting biofilm formation. Consequently, metal NPs exhibit potent antibacterial properties, which make them promising avenue for periodontal therapy (Hosseini Hooshiar et al., 2024).

#### 5.1.1 GNSs

The small size of NPs can increase their reactivity and toxicity, leading to cytotoxicity, genotoxicity, reticuloendothelial system toxicity, and altered inflammatory responses. Ensuring the biological safety of nanomaterials is crucial to prevent potential harm to human health. Biocompatible materials such as GNSs are essential for mitigating these adverse reactions (Zhang S. et al., 2024). GNSs are less hazardous to mammalian cells because their antibacterial effects do not rely on ROS (Kiarashi et al., 2024). Their ease of synthesis, excellent biocompatibility, and high potential for functionalization make GNSs valuable for drug delivery, diagnosis, and therapy, including anti-inflammatory and antitumor applications (Zhou et al., 2021). In bone regeneration, GNSs promote osteogenic differentiation of mesenchymal stem cells, inhibit osteoclast formation, and accelerate bone formation (Zhang et al., 2021). They may influence cytoskeletal structures through endocytosis, thereby promoting osteogenic differentiation via autophagy activation (Zhang et al., 2021). GNSs can also facilitate alveolar bone defect repair using cell sheet technology and modulate macrophage phenotypes to support periodontal tissue regeneration (Ni et al., 2019; Zhang et al., 2021). As carriers, GNSs protect polypeptides from protease degradation and facilitate their cellular entry via receptor-mediated endocytosis (Wang S. et al., 2018). Human β-defensin 3 (hBD3), a broad-spectrum antibacterial peptide (Harder et al., 2001), promotes osteogenesis in periodontal tissues (Zhu et al., 2017). GNSs combined with hBD3 can inhibit the secretion of receptor activator of nuclear factor kappa-B ligand (RANKL) and increase OPG expression, potentially reducing alveolar bone resorption. Advances in nanomaterials and tissue engineering have suggested that combining GNSs with hBD3 can effectively treat periodontitis, representing a novel approach to periodontal therapy (Zhou et al., 2021).

#### 5.1.2 AgNPs

AgNPs are highly effective antibacterial agents against both gram-negative and gram-positive bacteria (Siddiqi et al., 2018; Yin et al., 2020). They exert antibacterial effects by increasing the membrane permeability and inducing ROS production through intracellular uptake (Liang et al., 2023). Despite their promise, the clinical use of AgNPs is limited due to toxicity concerns, such as silver accumulation in organs, particularly the liver and spleen (Yin et al., 2020), and oxidative stress and cellular injury from increased ROS production (Siddiqi et al., 2018; Palacios-Hernandez et al., 2020). Ebselen can mitigate these effects by increasing glutathione and superoxide dismutase 2 levels, reducing AgNP-induced ROS, and showing no significant cytotoxicity at concentrations up to 20 μg/mL in human gingival fibroblasts. Combining AgNPs with ebselen enhances their bactericidal efficiency, showing significant effects against planktonic *P. g* and *F. n* and demonstrating synergistic anti-biofilm activity on both mono- and multi-species biofilms (Liang et al., 2023). Silver-based biomaterials (AgBMs) exhibit several antimicrobial properties, including the disruption of bacterial cell membranes, enzymes and proteins, as well as damaging genetic material. These properties make AgBMs effective antibacterial agents while maintaining relatively low toxicity. They have been explored for various dental applications including implant coatings, periodontal plaque control, caries prevention, root canal sterilization, denture additives, and anti-inflammatory materials for oral and maxillofacial surgery (Hosseini Hooshiar et al., 2024).

#### 5.1.3 Copper sulfide (CuS) NPs

CuS, a metal sulfide semiconductor, has garnered significant attention due to its excellent photodynamic and photothermal properties under near-infrared light irradiation (Mo et al., 2022; Zhang et al., 2023). CuS-based NPs demonstrate strong antibacterial properties and promote wound healing (Liang and Yan, 2019; Wang X. et al., 2021). Yu et al. developed a novel protease-loaded CuS NP that combined the features of PDT, PTT, and protease activity. The experimental results confirmed the photothermal activity and the capacity of the NPs to generate ROS, which formed the basis for their antibacterial function. The CuS@A NPs exhibited high antimicrobial activity against *F*.*n* and its biofilms. *In vitro* assays demonstrated proper hemo- and cyto-compatibility. In a rat periodontitis model, CuS-based NPs effectively inhibited bone resorption and alleviated inflammation, indicating their potential as promising materials for managing periodontitis (Gao et al., 2023).

### 5.2 NZs used in periodontitis treatment

ROS generated by bacterial infection and autologous inflammatory tissues play crucial roles in the progression of periodontitis. Consequently, the elimination of excess intracellular ROS is a feasible approach for anti-inflammatory treatment. NZs have shown promising application in treating periodontal diseases by maintaining the intracellular redox balance and protecting cells from oxidative damage (Hosseini Hooshiar et al., 2024). NZs usually comprise metal oxide NPs, noble metal nanomaterials, and other substances that display four main catalytic activities: SOD, CAT, OXD, and POD. With the rapid advancement of nanomedicines, scientists have enhanced the catalytic qualities of NZs, enabling them to interact with specific target molecules in a selective and effective manner. NZs are primarily composed of metal‐based NZs, metal oxide/sulfide‐based NZs, carbon‐based NZs, MOFs, and single-atom nanozymes (SAzymes) (Hosseini Hooshiar et al., 2024) (**Figure 6**). We provide a comprehensive summary of the application of these NZs in the treatment of periodontitis.

#### 5.2.1 Metal‐based NZs

NZs composed of noble metals, such as platinum, silver, and gold, exhibit significant catalytic activity, aiding bacterial adhesion and cell membrane disruption. Their inherent OXD-like and POD-like properties induce intracellular ROS production, effectively kill bacteria, and promote wound healing (Zheng et al., 2018). Recent research has underscored the challenges of managing pathogenic biofilms, such as those from *F*.*n* in periodontitis, with traditional treatments, owing to bleeding, drug resistance, and limited efficacy. NZs represent a novel solution. For example, a bimetallic cluster zyme with enhanced POD-like activity and glucose oxidase exhibited outstanding biocompatibility and antibacterial characteristics. This clusterzyme converts glucose into toxic -OH, effectively inhibiting and eliminating *F.n* biofilm *in vivo*. Animal studies demonstrated that bimetallic cluster zyme effectively treated periodontitis in rats, reduced inflammation, and promoted the regeneration of periodontal tissue (Wang et al., 2023). Another study developed a TM/BHT/CuTA hydrogel system comprising Cu-based NZs (Cu-tannic acid coordination nanosheets, CuTA NSs) and a triglycerol monostearate/2,6-di-tert-butyl-4-methylphenol (TM/BHT) hydrogel. This system retains inflammatory sites via electrostatic adsorption and hydrolyzes them in response to increased MMPs, enabling the on-demand release of CuTA NZ. The released CuTA NZ exhibits antibacterial and anti-plaque properties, scavenges ROS by simulating SOD and CAT processes, and regulates the shift of macrophage polarization from M1 to M2 through the Nrf2/NF-κB signaling pathway, reducing inflammation and promoting tissue regeneration (Xu et al., 2023). In addition, FeSN, a histidine-doped FeS_2_ with high POD-like activity, demonstrated potent antibacterial activity, low cytotoxicity, and high biocompatibility. The catalytic efficiency of FeSN was approximately 30 times greater than that of FeS_2_. In the presence of H_2_O_2_, FeSN demonstrated strong antibacterial activity against *F*.*n*, increased OXD coenzyme levels, and decreased GR and ATP levels in bacterial cells. It effectively reduced biofilm development, inflammation, and loss of alveolar bone in a rodent model (Shen et al., 2023). These studies highlighted the potential of metal-based NZs for treating bacterial infections and periodontal diseases.

#### 5.2.2 Metal oxide/sulfide‐based NZs

CeO_2_ NPs exemplify a biological catalyst with high POD-like activity owing to the reversible redox transition between Ce^4 +^ and Ce^3 +^ ions. This transition efficiently facilitated the CeO–H_2_O_2_ ROS system. The high redox potential and surface-rich oxygen vacancies of nanoceria confer various enzymatic activities, such as those of SOD, CAT, POD, and OXD. These properties enable CeO_2_ NPs to mitigate inflammation through their antioxidant properties (Hosseini Hooshiar et al., 2024). Researchers have enhanced the biocompatibility and dispersion of CeO_2_ NPs by loading them onto mesoporous silica and modifying them with polyethylene glycol. This formulation protects PDLSCs from oxidative stress induced by periodontitis by modulating ROS levels and promoting osteogenic development (Ren et al., 2021; Cai et al., 2022). CeO_2_ NPs with strong CAT- and SOD-like activities demonstrated significant anti-inflammatory and antioxidant effects. CeO_2_ NPs were tested as *in situ* injections for managing periodontitis. Both *in vivo* and *in vitro* studies showed that CeO_2_ NPs effectively neutralize ROS and suppress inflammatory responses by impeding the MAPK–NFκB signaling pathway. In rodent models, CeO_2_ NPs notably decreased alveolar bone resorption, reduced osteoclast activity and inflammation, and supported tissue regeneration (Yu et al., 2022). In summary, CeO_2_ NPs exhibit significant potential for clinical application in treating periodontitis. Their ability to scavenge ROS, inhibit inflammatory pathways, and promote tissue regeneration makes them valuable in managing oxidative stress-induced inflammatory diseases (Yu et al., 2022).

#### 5.2.3 Carbon‐based NZs

Carbon-based nanomaterials, including CDs, carbon nanotubes (CNTs), graphene and its derivatives, carbon nitrides, and fullerenes, are extensively used in the biomedical field due to their biocompatibility, physicochemical properties, and enzyme-mimicking abilities. Wang et al. produced a range of oxidized CNTs (o-CNTs) with a high concentration of oxidized groups, which exhibited superior POD-like activity across a broad pH range. The carbonyl groups on the surface of the o-CNTs serve as active catalytic centers, whereas the carboxyl and hydroxyl groups act as competitive sites. The carboxyl group, owing to its inherent negative charge and tendency to form hydrogen bonds, exhibited a greater inhibition of the catalytic activity than the hydroxyl group. Owing to their enhanced POD-like activity and unique surface properties, o-CNTs have great potential for various biomedical applications (Wang H. et al., 2018; Hosseini Hooshiar et al., 2024).

#### 5.2.4 MOFs

MOFs are crystalline and porous structures formed by organic linkers bridging metallic nodes and demonstrate outstanding mechanical and chemical characteristics. Recently, MOF-based NZs have proven to be highly promising for biological catalysis and sensing due to their versatile structures, high activities, and robust stabilities. MOFs provide a versatile platform for developing novel NZs with built-in catalytic properties for the treatment and diagnosis of bacterial infections. Ongoing research has led to the development of catalytic systems based on MOF and single atoms, which offer greater potential owing to their three dimensional porous structures and increased number of catalytic sites. Single-atom catalytic systems exhibit higher catalytic activity and lower metal consumption (Ali et al., 2021). In one study, researchers have developed an injectable ointment featuring potent anti-biofilm properties and excellent biocompatibility by employing a single-atom catalysis system based on MOFs. Specifically, they utilized a porphyrin-based MOF (PCN-222) integrated with Pt single atoms. Incorporating single metal atoms such as Pt, Au, Cu, and Ru into PCN-222 improves its OXD-like activity by lowering the adsorption and activation energies of O_2_. The resulting PCN-222-Pt spontaneously generated ROS and exhibited strong OXD-like- and POD-like activities. It showed remarkable anti-biofilm efficacy, achieving 98.69% inhibition against *S. aureus* biofilm and 99.91% inhibition against *E. coli* biofilm *in vitro* within just 1 hour. Compared to the clinically prescribed periocline, the injectable PCN-222-Pt ointment demonstrated a lower bone degradation rate, healthier periodontal tissue, and reduced inflammation in the treatment of biofilm-induced periodontitis. This offers a fast, efficient, non-invasive, and practical approach for treating periodontitis without antibiotics (Yu et al., 2021).

#### 5.2.5 SAzymes

NZs are extensively studied nanomaterials that exhibit enzymatic activity. However, challenges such as insufficient substrate selectivity, complex composition, and a low density of active sites have limited their widespread application. Enzymes featuring atomically dispersed active sites, which are known for their exceptional performance, have been used in the field of catalysis. SAzymes are highly regarded by researchers for their optimal atom utilization, cost-effectiveness, well-defined coordination structures, and distinct active sites (Han et al., 2023). They have become a focal point in NZ research because of their high activity, stability, and cost-effectiveness, making them excellent alternatives to natural enzymes. In comparison to conventional NZs, SAzymes exhibit superior catalytic activity and specificity owing to their enhanced atom utilization and accurately defined geometric and electronic structures (Jiang et al., 2023).

## 6 Prospects for NPs-targeted enzymes in periodontitis diagnosis and treatment

Non-invasive biomarkers for periodontitis are measured in the saliva or GCF. Saliva represents the general condition of the mouth rather than individual teeth, reducing its specificity. GCF provides more precise detection, but its limited volume (<2 μL) complicates the analysis of multiple biomarkers (He et al., 2021; Hooshiar et al., 2024). No single marker currently meets all criteria for evaluating the periodontium. Enzyme research may focus on developing “marker packages” and designing ideal chairside diagnostic tests (Hooshiar et al., 2024). A challenge in enzyme research is correlating protein quantity with pathophysiology because enzymatic activity is not always proportional to concentration. This necessitates activity-based readouts, rather than concentration measurements, to link specific enzymes to tissue destruction (Koller et al., 2021). MS and immunoassays, such as ELISA and immunofluorometric assay (IFMA), can identify and quantify salivary biomarkers but fall short in determining enzymatic activity. Accurate enzyme activity profiling requires western blot or other enzymatic catalysis tests, which are time-consuming, costly, and unsuitable for routine clinical diagnosis (Silbereisen et al., 2020). Biosensors and fluorescent probes offer numerous advantages, such as high sensitivity, selectivity, reproducibility, affordability, minimal sample needs, and user-friendliness. Some nanoprobes have detection limits as low as femtomoles, offering significant sensitivity compared to conventional techniques. Despite this promise, challenges remain regarding their clinical implementation (Hooshiar et al., 2024). Further studies are needed to refine these methods and establish detection limits for earlier diagnosis (Koller et al., 2021).

A predictive medicine approach for early-stage periodontitis prevention is a leading research direction, requiring advanced monitoring and analysis measures to understand the underlying molecular mechanisms (Pei et al., 2020). A combination of nanomaterials with artificial intelligence (AI) is currently employed to achieve multiplexing. Advanced NP-based platforms have been created to detect biomarkers linked to both infectious and non-infectious diseases, resulting in more precise and effective diagnostic procedures (Thwala et al., 2023). The future of enzyme-based diagnostics for periodontitis lies in overcoming the existing challenges through continued research and technological advancements. Integrating nanomaterials, AI, and improved biosensing techniques has the potential to revolutionize the early diagnosis and treatment of periodontal disease. The application of nanomaterial-targeted enzymes for the diagnosis and treatment of periodontitis presents both promising and challenging opportunities. Addressing these challenges involves the following steps.

1. Enhancing material performance: The efficacy of nanomaterials in targeting enzymes significantly depends on their intrinsic properties. Optimizing the surface chemistry, size, and shape of nanomaterials can increase their binding affinity and specificity for enzymes involved in periodontitis, while also helping to control their toxicity and immune responses. Future research should focus on the development of multifunctional nanomaterials that can simultaneously perform diagnostic and therapeutic functions (theranostics) and potentially revolutionize periodontal treatment strategies (Hooshiar et al., 2024).
2. Improving detection technologies: Detecting enzymes at low concentrations requires amplification techniques to enhance the detection signals. Advances in nanotechnology and biosensing methods have offered potential solutions. Techniques such as surface-enhanced Raman scattering, FRET (Lee et al., 2016; Yuan et al., 2022), and electrochemical sensing can significantly amplify detection signals, enabling the identification of enzymes at much lower concentrations. The integration of advanced detection technologies with nanomaterials provides highly sensitive and specific diagnostic tools for periodontitis.
3. Achieving lower detection limits: Achieving lower detection limits for enzymes is critical for the early diagnosis and effective treatment of periodontitis. Strategies to lower the detection limits include developing nanomaterials with higher surface-area-to-volume ratios, enhancing interactions with target enzymes (Mok et al., 2020), using signal amplification techniques, and optimizing assay conditions. Nanobiosensors have attracted significant attention because of their ability to detect a broad range of analytes at extremely low concentrations. As this field advances, new POC diagnostic methods that are ultrasensitive, affordable, robust, and reliable are anticipated to emerge (Hooshiar et al., 2024).
4. Integration of artificial intelligence: The integration of AI with nanomaterials offers a transformative approach for the diagnosis and treatment of periodontitis. AI algorithms can be used to analyze complex datasets generated from nanomaterial-based sensors, providing insights into the patterns and levels of enzyme activity. Machine learning models can predict disease progression and treatment outcomes, thereby enabling personalized and targeted therapeutic interventions. Nanoenabled AI-supported smart optical biosensors have the potential to facilitate rapid point-of-care detection with real-time sensitivity, and portable capabilities (Taha et al., 2024). Combining AI with nanomaterials can also facilitate the development of smart diagnostic platforms that adapt to changes in the biological environment in real-time, ensuring more accurate and timely responses to periodontal diseases (Malik and Waheed, 2023).

The future of NP-targeted enzymes in the diagnosis and treatment of periodontitis is promising, with significant potential to overcome the current limitations. Continued interdisciplinary research and collaboration are essential for fully realizing the potential of these advanced technologies in clinical practice. Future research should focus on overcoming the limitations associated with the nanodental industry, conducting safety analyses, and integrating artificial intelligence for enhanced diagnostic and therapeutic applications.

## 7 Conclusion

Early diagnosis and prevention of periodontitis are crucial for improving periodontal health. First, we review various enzyme biomarkers associated with periodontitis, including microbial proteases, oxidative stress-related enzymes, and proteolytic enzymes. These biomarkers are valuable for assessing the different stages of periodontitis, identifying early disease, and evaluating treatment efficacy. The second part summarizes the mechanism of the NPs’ function. Finally, we review the applications of NPs in the diagnosis and treatment of periodontitis. The future of periodontitis research lies in the combination of AI technology and nanotechnology. This integration can create smart periodontal diagnostic platforms that target enzymes, thereby enabling dynamic monitoring, early prevention, and proactive treatment. This approach represents a burgeoning area of research that promises significant advancements.

## Glossary

PD: Periodontitis
RA: Rheumatoid Arthritis
TP: Temporal Pole
AMD: Age-Related Macular Degeneration
TICL: Toric Intraocular Collamer Lens
CEUS: Contrast-Enhanced Ultrasound
OS: Overall Survival
RFS: Recurrence-Free Survival
MD: Meniere’s Disease
KOA: Knee Osteoarthritis
ECM: Extracellular Matrix
*P.g*: *Porphyromonas gingivalis*
*T.d*: *Treponema denticola*
*F.n*: *Fusobacterium nucleatum*
*A.c*: *Actinobacillus actinomycetemcomitans*
DPP: Dipeptidyl Peptidase
NOX: Nicotinamide Adenine Dinucleotide Phosphate Oxidase
TAC: Total Antioxidant Capacity
SOD: Superoxide Dismutase
MPO: Myeloperoxidase
NE: Neutrophil Elastase
BGD: Beta-glucuronidase
PR3: Proteinase 3
PRTN3: Chymotrypsin-like Proteinase 3
PMNs: Polymorphonuclear Neutrophils
aMMP-8: Active Matrix Metalloproteinase-8
PoC: Point-of-Care
Hb: Hemoglobin
OPG: Osteoprotegerin
OA: Osteoarthritis
ALP: Alkaline Phosphatase
CuO NPs: Copper Oxide Nanoparticles
ELISA: Enzyme-Linked Immunosorbent Assay
GCF: Gingival Crevicular Fluid
GNP: Gold Nanoparticle
GNSs: Gold Nanostructures
HNE: Human Neutrophil Elastase
LSPR: Localized Surface Plasmon Resonance
POCT: Point-of-Care Testing
rGO: Reduced Graphene Oxide
UCNPs: Up-Converting Nanoparticles
AgNPs: Silver Nanoparticles
ROS: Reactive Oxygen Species
CuS: Copper Sulfide
PDT: Photodynamic Therapy
PTT: Photothermal Therapy
NPs: Nanoparticles
NZs: Nanozymes
OXD: Oxidase
CAT: Catalase
MMPs: Matrix Metalloproteinases
GR: Glutathione Reductase
ATP: Adenosine Triphosphate
CeO_2_ NPs: Cerium Oxide Nanoparticles
PDLSCs: Periodontal Ligament Stem Cells
CNTs: Carbon Nanotubes
CDs: Carbon Dots
MOFs: Metal-Organic Frameworks
PCN-222: Porphyrin Metal-Organic Framework
SAzymes: Single-Atom Nanozymes
CNTs: Carbon Nanotubes.
